# Band Unfolding in Finite Nanostructures: Visualizing Dirac, Spin–Valley, and Rashba Features

**DOI:** 10.1021/acs.nanolett.5c04721

**Published:** 2025-12-10

**Authors:** Naoya Yamaguchi, Sefty Yunitasari, Wardah Amalia, Chi-Cheng Lee, Taisuke Ozaki, Fumiyuki Ishii

**Affiliations:** † Nanomaterials Research Institute (NanoMaRi), 12858 Kanazawa University, Kakuma-machi, Kanazawa 920-1192, Japan; ‡ Graduate School of Natural Science and Technology, 12858 Kanazawa University, Kakuma-machi, Kanazawa 920-1192, Japan; ¶ Department of Physics, 34886 Tamkang University, Tamsui, New Taipei 251301, Taiwan; § Institute for Solid State Physics, The University of Tokyo, Kashiwa-no-ha, Kashiwa 277-8581, Japan

**Keywords:** band dispersion, graphene flake, bent flake, tungsten disulfide flake, spin splitting, first-principles calculation

## Abstract

Nanomaterials possess unique electronic properties distinct from bulk systems, and spatially resolved techniques such as nano-ARPES now allow for local band structure measurements at the nanoscale. However, theoretical tools for interpreting band dispersion in finite, aperiodic systems remain limited. Here, we propose a “giant molecule band unfolding” (GMBU) procedure that enables the extraction of band dispersion from molecular orbital levels of finite systems without assuming periodic boundary conditions. Using first-principles calculations for graphene, tungsten disulfide, and bismuth/silver surface alloy nanoflakes, we successfully reproduced the characteristic band structures of Dirac cones, spin-valley locking, and Rashba spin splitting, respectively. Our spin-resolved formulation visualizes spin textures and provides an efficient framework for analyzing spintronic and valleytronic properties. GMBU enabled visualization of band dispersion, even when nanoflakes were bent. The method bridges discrete and continuous electronic descriptions and is applicable across dimensionalities and symmetry classes, offering new possibilities for understanding and designing functional nanoscale materials.

## Graphical abstract

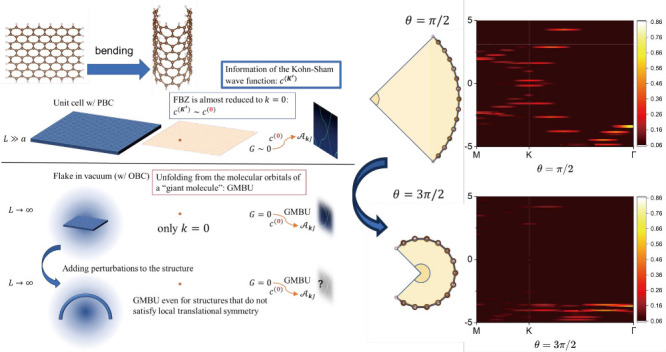

Nanomaterials, unlike bulk materials, possess unique electronic structures and are an important group of materials for advanced technologies, such as electronic devices and quantum devices. Furthermore, two-dimensional nanomaterials have the potential to exhibit new physical properties and multifunctionality through the formation of heterostructures. In order to control nanomaterials, sample preparation techniques are essential, but the development of measurement techniques is also indispensable. Angle-resolved photoemission spectroscopy (ARPES) is an experimental technique for analyzing the electronic structure of materials. Band structure analysis is important and useful for discovering new materials and understanding the properties of existing materials. Recently, attempts have been made to add spatial resolution to ARPES, and devices with spatial resolution ranging from micrometers to nanometers (e.g., μ-ARPES, nano-ARPES) have been developed. In order to design ultrafine nanoelectronic devices, it is important to control domain structures and local electronic structures using measurement techniques. Nano-ARPES is used to observe local electronic structures in local domains in polycrystals and nanomaterials.^1–8^ In particular, observations have been made in two-dimensional materials such as polycrystalline graphene^9–14^ and van der Waals (vdW) heterostructures.^15–19^ vdW heterostructures are expected to exhibit unique electronic structures not observed in conventional crystals, as they allow for highly controlled atomic layer arrangements even in cases of lattice mismatch, and are actively studied.^20^ These nanomaterials with unique nanostructures hold promise for the design of functional materials and the creation of energy materials. The importance of the band structure analysis of nanomaterials is increasingly recognized from the perspectives of elucidating the origin of material properties and material design. The spatial resolution of nano-ARPES is on the order of 100 nm. If ARPES reaches the nanoscale, it may become possible to actively handle the local electronic structures of nanomaterials. However, detailed analysis of the electronic structures of polycrystalline structures with multiple domains, such as band structure analysis in systems that do not satisfy periodic boundary conditions, has not yet been established by using first-principles calculations.

In this letter, we proposed an idea of a “giant molecule band unfolding (GMBU)” procedure that uses the band unfolding method (see Supporting Information, references 1−10) for giant molecule models to investigate the band dispersion extracted from finite systems such as flakes. We performed first-principles calculations based on the density functional theory (DFT) for graphene and transition metal dichalcogenide (TMD) nanoflakes to validate it. Two-dimensional TMD materials have a variety of potential applications and are also known for their valley degree of freedom. The computational results almost reproduced the characteristic bands of graphene and WS_2_ sheets. Furthermore, for bismuth/silver surface alloys, the Rashba spin splitting was also reproduced, even in the flake system. Our demonstration suggests that with a giant molecule to model a finite system extracted from an arbitrary system the band unfolding method may extract the band dispersion of any system. The procedure will be effective and convenient even for cases of aperiodic systems, and it will stimulate comprehensive understanding of the electronic structures in finite systems with local periodicity. The spin-resolved band unfolding will aid in the development of novel nanomaterials for next-generation nanoelectronics utilizing spin and valley properties.

Our proposed GMBU procedure applies the band unfolding method to finite systems by extracting band dispersion information from molecular orbital (MO) energy levels without relying on periodic boundary conditions. As shown in Figure 1(a), when a supercell is formed, the first Brillouin zone is reduced. However, the band dispersion for the supercell computational model can be restored to the image in the first Brillouin zone of the original unit cell. If the lattice constant of the supercell is sufficiently larger than that of the original cell, then the first Brillouin zone is reduced to the point *k* = 0. This suggests that the band structure of the original cell can be restored by unfolding the band structure at the point *k* = 0. When one cuts out that supercell to create a flake, the first Brillouin zone will be only the point *k* = 0, and one can expect that the band dispersion for the supercell computational model can be restored to the image in the first Brillouin zone of the original cell. Furthermore, by applying perturbations to the structure, such as bending the cut-out structure, the local translational symmetry is no longer satisfied. However, how the electronic structure is affected by the perturbation is also important from the perspective of nanostructure device design. The band unfolding in a system that does not satisfy translational symmetry can be attempted using the relative position vectors between atoms instead of the lattice vectors, and indeed, our GMBU procedure makes this possible (Figure 1(b)); the spectral weight in GMBU is

$$A_{kJ}^{\mathrm{GMBU}}\equiv \gamma \underset{MN}{\sum }c_{MJ}^{\left(0\right)*}c_{NJ}^{\left(0\right)}\underset{N^{\prime }R}{\sum }\delta_{n\left(N\right),n\left(N^{\prime }\right)}e^{ik\cdot \left(R+\tau \left(N^{\prime }\right)-\tau \left(N\right)\right)}\left\langle \phi_{M}^{0}|\phi_{N^{\prime }}^{R}\right\rangle$$ 1

where *c*_*NJ*_^(**0**)^ is the eigenvector corresponding to the Kohn–Sham wave function, and τ­(*N*) is the position of an atom that the *N*-th AO belongs to (see also Section 1 of Supporting Information). Because the calculations of the GMBU procedure do not depend directly on the lattices but only on the atomic positions, it can be applied straightforwardly and efficiently, even to systems with large distortions and curved shapes that cannot be handled by conventional band unfolding methods. In short, the GMBU procedure evaluates *k*-dependent spectral weights, allowing visualization of band structures in finite nanostructures modeled as large molecules. Since the GMBU procedure does not impose the condition of local translational symmetry, it is a more general method than conventional band unfolding methods. The procedure is computationally efficient and applicable to aperiodic systems, providing a bridge between discrete MO levels and continuous band structures (see also Section 1 of the Supporting Information).

**Figure 1:**
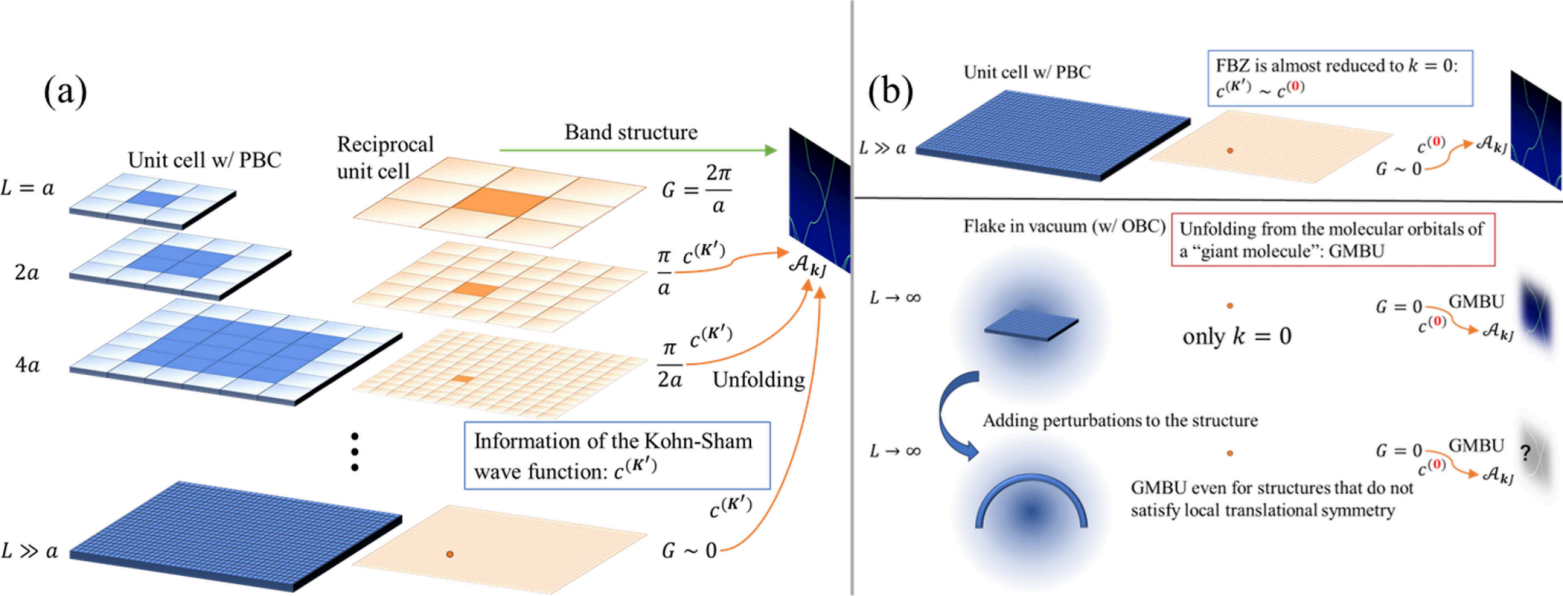
Schematic of band unfolding and giant molecule band unfolding (GMBU): (a) Supercell transformation and band unfolding. The dark blue and dark orange cells represent the unit cells under the periodic boundary condition (PBC) in real space and reciprocal space, respectively. Band unfolding allows the band dispersion of the primitive cell to be reconstructed by estimating the spectral weights $A_{kJ}$ from information on the Kohn–Sham wave function; (b) GMBU and its applicability. Band unfolding for flakes cut out as giant molecules, under the open boundary condition (OBC), can be performed in the same way as that for bulk periodic cells. Although local translational symmetry is no longer satisfied when the flake is bent, it is still possible to extract band dispersion using GMBU.

We validated the GMBU procedure to extract the band dispersion in finite systems by applying it to graphene nanoflakes. First, we prepared computational models for graphene nanoflakes with different numbers of carbon rings composing a honeycomb structure shown in Figure 2(b), where the numbers of atoms are 16, 30, 48, and 96 for 2 × 2, 3 × 3, 4 × 4, and 6 × 6 ring cases, respectively. After the geometry optimization for all the atoms, the averages of the bond length between carbon atoms in 2 × 2, 3 × 3, 4 × 4, and 6 × 6 models were 1.39 Å, 1.40 Å, 1.41 Å, and 1.42 Å, respectively, and the average bond length in the 6 × 6 case corresponds to the lattice constant of 2.46 Å, consistent with the experimental value of graphene. Then, we investigated the flake size dependence on the spectral weight of the band dispersion for graphene nanoflake models indicated in Figure 2(b). The calculated spectral weights are shown in Figure 2(a). The energy band appeared based on the MO energy levels, and the allowed and forbidden bands arose due to how high MO energy levels were located (see also Section 7 of the Supporting Information). As the flake size became larger, the allowed band domains were extended, while the forbidden ones were shrunk. The 6 × 6 case almost reproduced the band dispersion of graphene in the case of the bulk without the edges except the forbidden band domains. In addition, nearly flat bands were also found, and they crossed the Fermi level.

**Figure 2:**
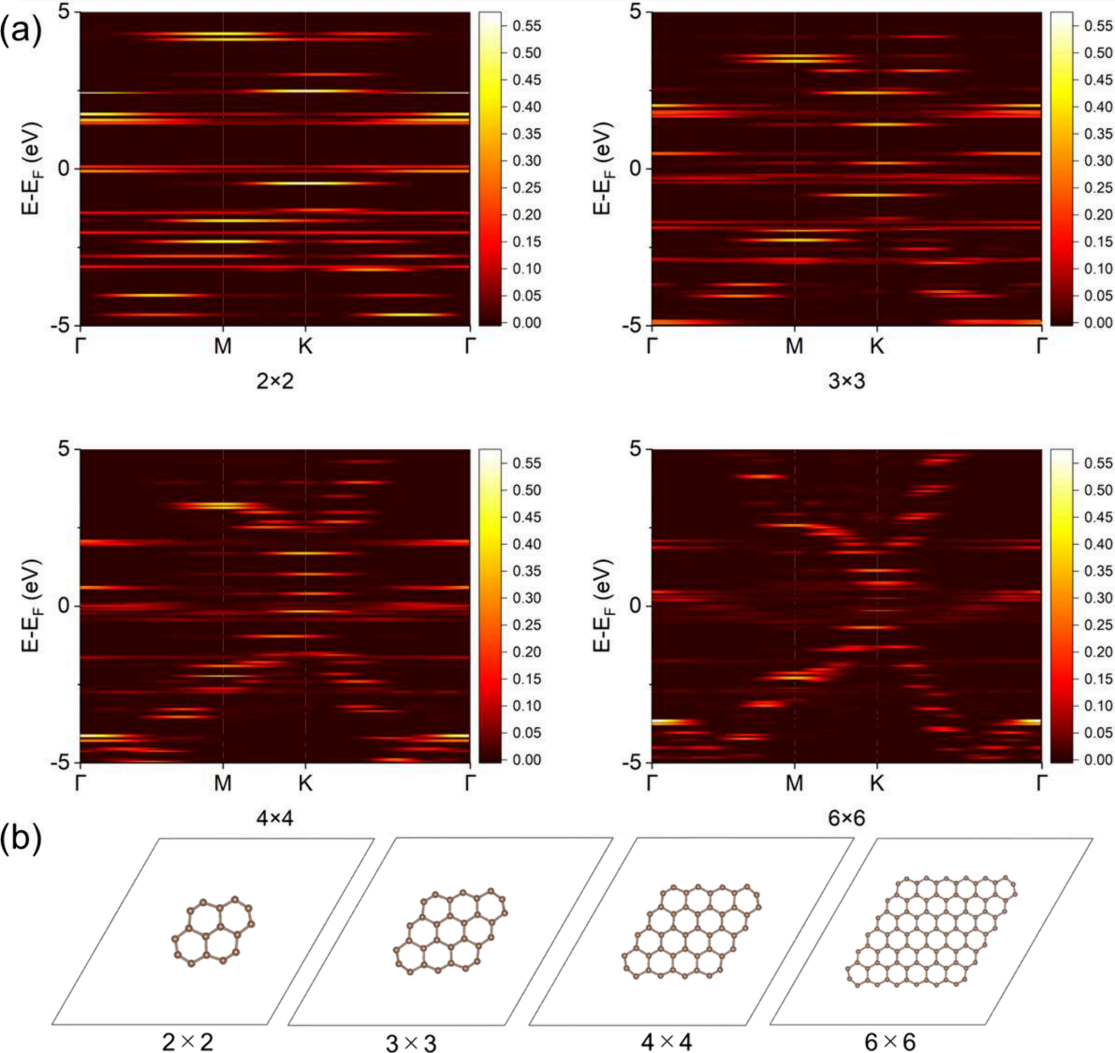
(a) Band dispersion extracted from graphene nanoflake models of 2 × 2, 3 × 3, 4 × 4, and 6 × 6 rings, where the color in the heatmap stands for the spectral weight. The *k*-path connects special *k*-points of Γ­(0, 0, 0), M­((2π/*a*)­(1/2, 0, 0)), and K­((2π/*a*)­(2/3, 1/3, 0)), where *a* is the lattice constant of pristine graphene. The Fermi level is set to the origin of the energy axis. The background is colored. (b) Top view of graphene nanoflake models of 2 × 2, 3 × 3, 4 × 4, and 6 × 6 rings. The balls and sticks represent carbon atoms and bonds between them, respectively. The rhombus represents the unit cell.

We also addressed a 15 × 15 nm flake to visualize the clear linear relation in the Dirac cone. We modeled graphene with hydrogen-terminated edges to prevent the edge states induced by the dangling bonds (see also Section 2 of the Supporting Information). Figure 3 shows the band structure of the 15 × 15 flake, and it showed the Dirac-cone-wise band dispersion clearly as expected in the limit of the infinitely large flake (i.e., pristine graphene without edges).

**Figure 3:**
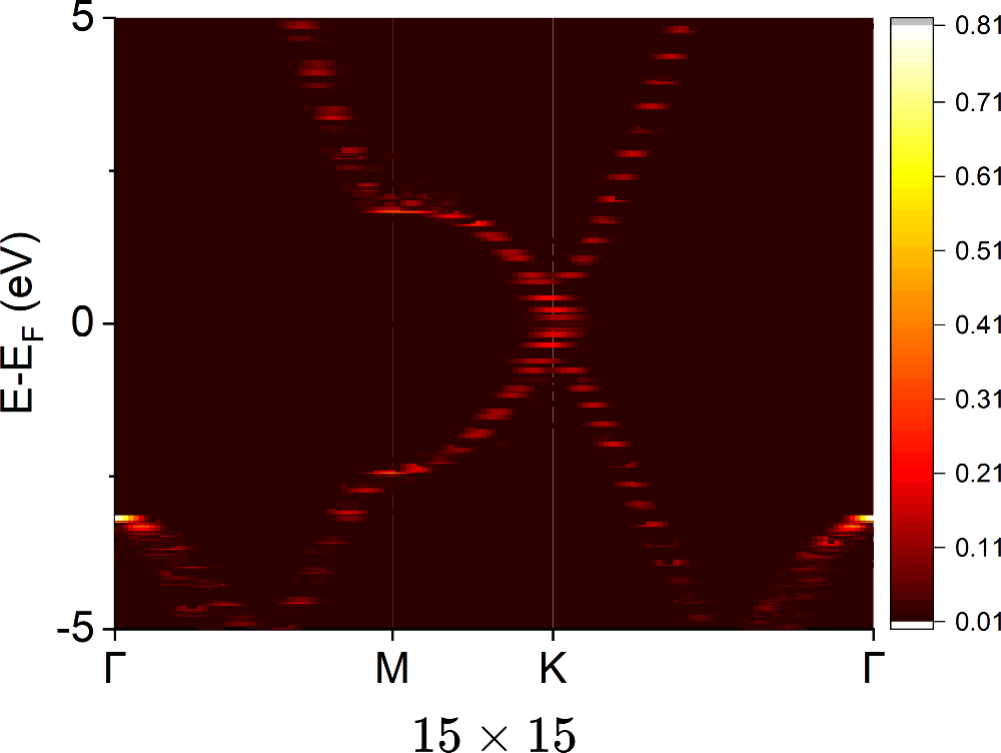
Band dispersion extracted from a graphene flake model of 15 × 15 rings. The *k*-path connects special *k*-points of Γ­(0, 0, 0), M­((2π/*a*)­(1/2, 0, 0)), and K­((2π/*a*)­(2/3, 1/3, 0)), where *a* is the lattice constant of pristine graphene. The color in the heatmap stands for the spectral weight, and weights less than 0.01 are omitted. The Fermi level is set to the origin of the energy axis. The background is colored.

Through the GMBU procedure, the band dispersion in the graphene nanoflake was visualized, and the flake size dependence suggested that if the flake size was large enough, the complete band dispersion of graphene suppressing the forbidden band domains could be realized. It was suggested that the existence of dangling bonds gave partially filled occupation for the nearly flat bands crossing the Fermi level. The dangling bonds made flat bands in the edge domain, and the flat bands correspond to edge states based on localized electrons. It is obvious that these nearly flat bands and flat bands originated from the finite size effect at edges since no such bands arose in an infinite graphene system, although the hydrogen termination reduces such flat bands as in the 15 × 15 case. The GMBU procedure supplied the intuitive description that the edge bands covered the bulk bands in the band dispersion picture. We demonstrated that the GMBU procedure enables us to find the band dispersion in finite systems with a giant molecule model and band unfolding. Moreover, the GMBU procedure can utilize the band unfolding method to elevate the representation dimension in the momentum space for the electronic structures of solids.

Twisted bilayer graphene was reported to exhibit a new superconducting phenomenon.^20^ Graphene can form layered structures via van der Waals (vdW) interactions, but vdW heterostructures composed of vdW interactions have also attracted attention and have been used in the design of electronic devices.^21^ These materials are considered as candidates for high-functionality materials due to their flexibility,^22,23^ such as the ability to change the electronic structure by changing the thickness of the sheet.^24–26^ The band unfolding method is effective for analyzing band dispersion affected by defects and impurities even in two-dimensional materials.^27,28^ However, there is atomic layer incommensurability in vdW materials, which makes compact modeling difficult.^29–36^ Therefore, our proposed GMBU method does not need to change the consistency of the simulation cell, which may solve the computational problem of having to deal with large, approximate crystals.

As with graphene, we also applied the GMBU procedure to a WS_2_ nanoflake. WS_2_ includes heavy elements, that is, tungsten, and large spin–orbit splitting is expected among TMDs. A WS_2_ nanoflake was modeled for 9 × 9 rings (Figure 4(d); see also Section 3 of the Supporting Information). We extended the GMBU procedure to visualize the spin-resolved band dispersion. We defined spin-α (α = *x*, *y*, *z*)-polarized spectral weight as ${\left\langle \sigma_{\alpha }\right\rangle }_{kJ}=\underset{j}{\sum }\left\langle \psi_{j}^{\left(k\right)}\left|\sigma_{\alpha }Â\left(\epsilon_{0J}\right)\right|\psi_{j}^{\left(k\right)}\right\rangle /\delta \left(0\right)=\gamma {\left(c^{\left(0\right)†}\left(\sigma_{\alpha }⊗S^{\left(k\right)}\right)c^{\left(0\right)}\right)}_{JJ}=A_{kJ}\cdot \left({\left\langle \sigma_{\alpha }\right\rangle }_{kJ}/A_{kJ}\right)$ where σ_α_ is the α-component of the Pauli matrices (see also Section 1 of the Supporting Information). As shown in Figure 4, the overall band dispersion is consistent with that of the pristine case, except for the influence of edges (Figure 4(a,c)). The spectra with spin −*z* and +*z* polarization around the valence band top at the K-point were split into upper and lower parts, respectively, and the spin-valley locking was clearly visualized (Figure 4(b)), while the spin degenerate bands appear around the Γ- and M-points. To illustrate the application of the in-plane spin components as well, we also applied the GMBU procedure to a Bi/Ag(111)- $\left(\sqrt{3}\times \sqrt{3}\right)$ *R*30° surface alloy nanoflake.^37,38^ The bismuth/silver surface alloy exhibits giant Rashba spin splitting,^39^ which is caused by the spin–orbit interaction and spatial inversion symmetry breaking along the out-of-plane direction. The Rashba spin splitting is a spin splitting in which the spin direction is in the in-plane direction. A large Rashba spin splitting around the Γ-point near the Fermi level was also confirmed even in the case of a flake (see Sections 4 and 5 of the Supporting Information).

**Figure 4:**
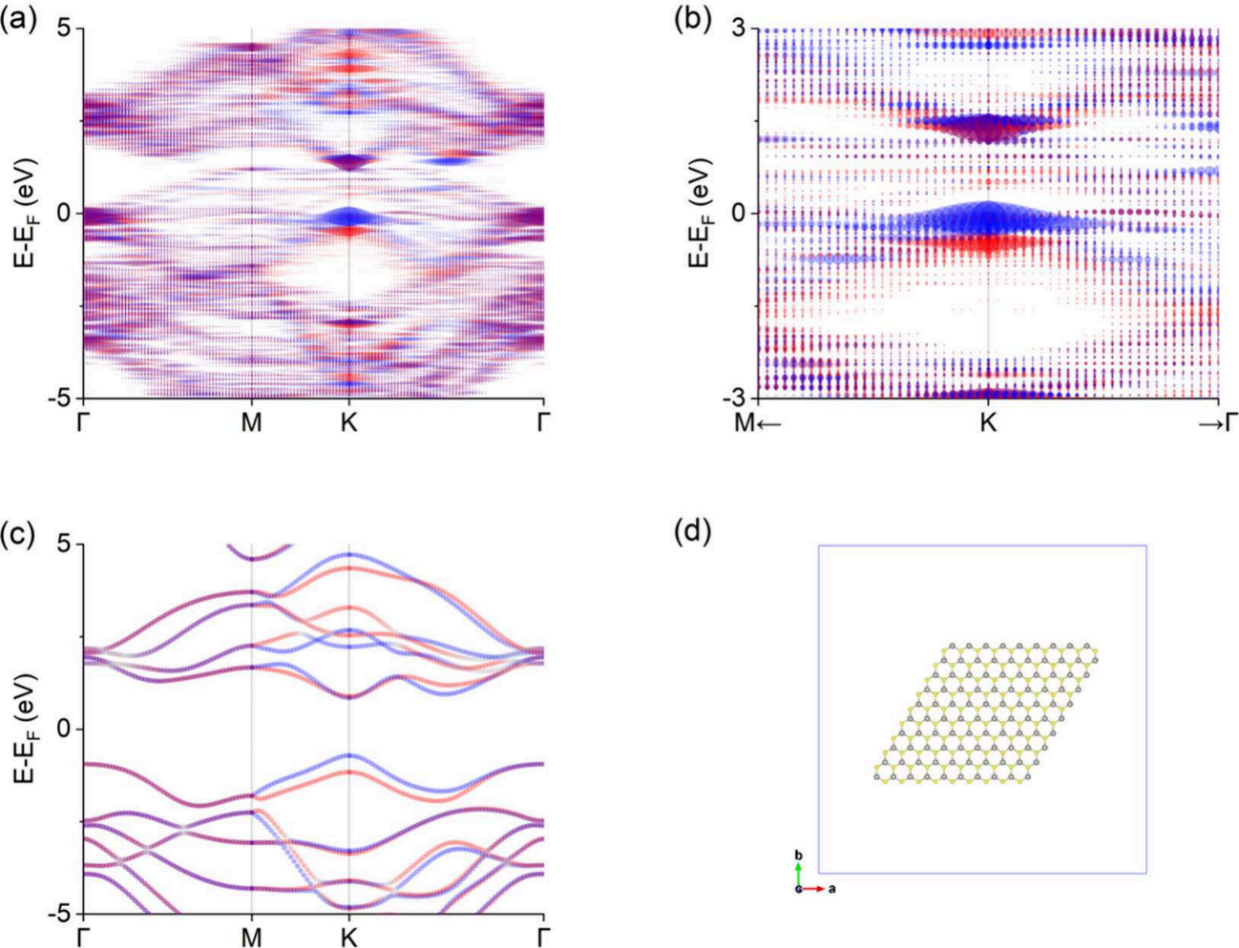
(a) Spin-resolved band dispersion extracted from a WS_2_ flake model of 9 × 9 rings. The *k*-path connects special *k*-points of Γ­(0, 0, 0), M­((2π/*a*_WS_2__)­(1/2, 0, 0)), and K­((2π/*a*_WS_2__)­(2/3, 1/3, 0)), where *a*_WS_2__ is the lattice constant of a pristine WS_2_ sheet. The blue and red circles represent the negative and positive signs of normalized spin-*z*-polarized spectral weights ${\left\langle \sigma_{z}\right\rangle }_{kJ}/A_{kJ}$ , respectively, and the radius of each circle reflects the magnitude of $A_{kJ}$ . The points at which $A_{kJ}$ is less than 0.01 are omitted. (b) Enlarged view of the vicinity of the K-point in (a). The horizontal axis is plotted within a distance of 0.3 Bohr^–1^ from the K-point. (c) Spin-resolved band dispersion of a pristine WS_2_ sheet as a comparison with (a). The Fermi level is set to the origin of the energy axis. (d) Top view of a WS_2_ nanoflake model of 9 × 9 rings. The gray and yellow balls represent tungsten and sulfur atoms, respectively, and sticks stand for bonds between atoms. The blue square represents the unit cell.

Finally, as a demonstration of the GMBU procedure’s extreme effectiveness, we examined the case of bent graphene nanoflakes. We extracted the band dispersions for bent graphene nanoflakes when the central angle of the arc viewed from the side was θ = 0, π/2, π, and 3π/2, and the results are shown in Figure 5. Up to θ = π/2, the characteristic Dirac cone-like spectral weight of graphene remains strongly present. However, at θ = π, this spectral weight weakens, and at θ = 3π/2, no strong spectral weight exists near the Fermi level. The degree of overlap of the p_*z*_ orbitals changed due to the curvature, resulting in observed variations in the Dirac cone-like spectral weight. Thus, bending nanoflakes causes even local translational symmetry to be lost, yet the GMBU effectively extracts the band dispersion.

**Figure 5:**
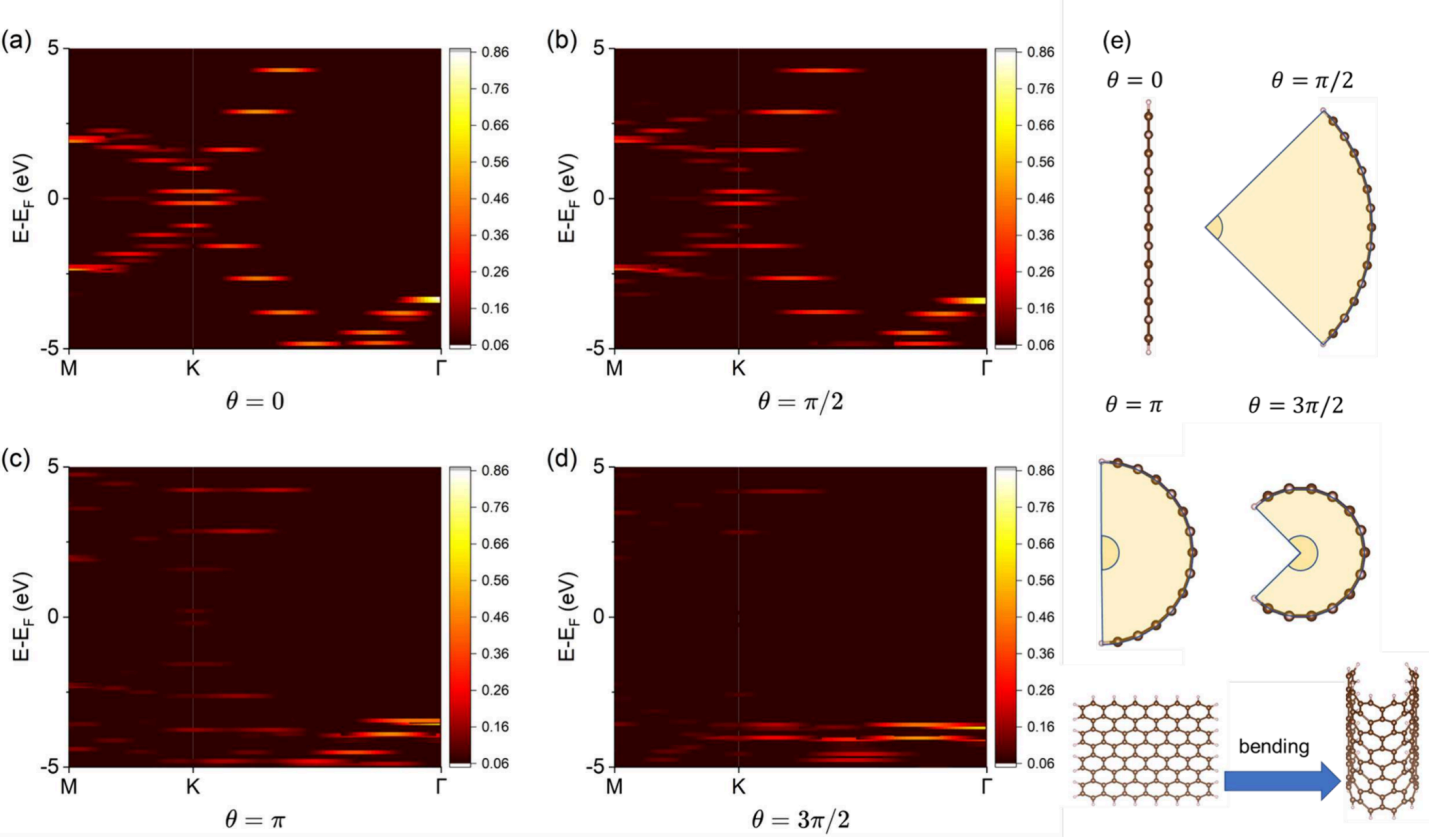
Band dispersion extracted from bent graphene nanoflakes for the central angles of (a) θ = 0, (b) θ = π/2, (c) θ = π, and (d) θ = 3π/2. The *k*-path connects special *k*-points of M­((2π/*a*)­(1/2, 0, 0)), K­((2π/*a*)­(2/3, 1/3, 0)), and Γ­(0, 0, 0), where *a* is the lattice constant of pristine graphene. The color in the heatmap stands for the spectral weight, and weight less than 0.06 is omitted. The Fermi level is set to the origin of the energy axis. The background is colored. (e) Side view of bent graphene nanoflakes for the central angles of the arc viewed from the side of θ = 0, θ = π/2, θ = π, and θ = 3π/2. The bent graphene was modeled by bending the square graphene nanoflakes shown in the top view. The dark brown and light brown balls represent carbon and hydrogen atoms, respectively, and sticks stand for bonds between atoms.

In the angle-resolved photoemission studies, spatial resolution techniques have been developed. Observations of the local and spatially resolved electronic structure with the energy dispersion relation in nanocrystals were achieved.^40–44^ In designing nanoelectronic devices, such a direct measurement would be essential to control the domain structure and the local electronic structure. Our models for graphene nanoflakes had a scale of less than 10 nm. For the WS_2_ flake, spin-valley locking was reproduced at flake sizes of a few nanometers, while the Rashba spin splitting was done for the Bi/Ag surface alloy flake. According to the results, dispersive band structures can arise within a spot area smaller than a resolution of a sub-micrometer scale achieved by the current nano-ARPES techniques. In addition, the finite size effect also appeared as the forbidden bands, as in the band dispersion. Our results implied that photoemission techniques with higher nanometer resolution are desirable and promising to nanoelectronics.

We have successfully extracted graphene, WS_2_, and Bi/Ag surface alloy nanoflake systems and reproduced the spin-resolved band dispersion of spin-valley locking of WS_2_ nanoflakes and the Rashba spin splitting of Bi/Ag surface alloys by adding a spin-resolved function to the band unfolding method. In fact, it has been reported that the Rashba effect manifests itself as a spin-charge conversion phenomenon even in aperiodic systems,^45–47^ and it is expected that the GMBU approach will enable the discovery of potential order in such electronic structures. The spin-resolved band unfolding method that we have developed was able to visualize the *x*-, *y*- and *z*-polarization cases in spin–orbit splitting, and it is expected to be useful for the analysis of materials that contribute to spintronics and valleytronics using spin and valley as information carriers. Furthermore, by demonstrating the applicability of GMBU nanoflakes when bent, we have shown a pathway for evaluating the electronic structure of material systems possessing flexible nanostructures—unconstrained by local translational symmetry—as band dispersion. The GMBU procedure may be expected to lead to the development of novel nanomaterials for next-generation nanoelectronics.

Although we discussed the application of the GMBU procedure to nanoflake systems, that is, two-dimensional cases, the GMBU procedure can be extended to one- or three-dimensional cases, such as finite chains and nanoparticles. Not only vdW materials but also other aperiodic systems can have the hidden band dispersion that is possible to analyze with the GMBU procedure. We stress that the GMBU procedure does not demand as large a simulation cell as the commensurate cell procedure in modeling aperiodic systems and gives novel applications for aperiodic systems before conventional band unfolding. Since our GMBU procedure is seamlessly connected to the conventional band unfolding method, it is possible to evaluate band dispersion from solids to nanomaterials such as supramolecules using a common representation. Recently, development of machine learning techniques for reconstructing band dispersion in solids has been carried out,^48^ and by utilizing the GMBU procedure, we may expect a further development in understanding comprehensive band dispersion reconstruction from periodic to aperiodic systems. As nano-ARPES techniques continue to develop with spatial resolutions currently reaching a few hundred nanometers, the GMBU procedure offers a complementary perspective for understanding local electronic structures in nanoscale materials.

## Supplementary Material



## Data Availability

The source code and data for giant molecule band unfolding (GMBU) can be found at https://doi.org/10.5281/zenodo.17815785.

## Notes

Theoretical details of the giant molecule band unfolding (GMBU), computational models, computational details, computational results of a bismuth/silver surface alloy flake with the Rashba spin splitting (PDF)
