# Supplementary material for: Band Unfolding in Finite Nanostructures: Visualizing Dirac, Spin–Valley, and Rashba Features
Source: Nano Lett. 2025 Dec 10;25(51):17725–32. doi: 10.1021/acs.nanolett.5c04721 (PMC12751115; doi:10.1021/acs.nanolett.5c04721)
Supplement: Supplementary file 1 [file nl5c04721_si_001.pdf]

Supplementary Information of "Band Unfolding in Finite Nanostructures: Visualizing Dirac, Spin–Valley, and Rashba Features"

Naoya Yamaguchi,*,† Sefty Yunitasari,‡ Wardah Amalia,‡ Chi-Cheng Lee,¶ Taisuke Ozaki,§ and Fumiyuki Ishii*,†

†*Nanomaterials Research Institute (NanoMaRi), Kanazawa University, Kakuma-machi, Kanazawa 920-1192, Japan*

‡*Graduate School of Natural Science and Technology, Kanazawa University, Kakuma-machi, Kanazawa 920-1192, Japan*

¶*Department of Physics, Tamkang University, Tamsui, New Taipei 251301, Taiwan*

§*Institute for Solid State Physics, The University of Tokyo, Kashiwa-no-ha, Kashiwa 277-8581, Japan*

E-mail: n-yamaguchi@cphys.s.kanazawa-u.ac.jp; ishii@cphys.s.kanazawa-u.ac.jp

1. Visualization of the band dispersion in finite systems

All the actual materials are finite, and one may consider the direct models with a large amount of atoms and the edges or surfaces in principle. In such a finite model for a crystal, hidden information of the band dispersion can be extracted from the molecular orbital (MO) energy levels of the model as a giant molecule. In the case, the periodic boundary condition for the crystal are not necessary, and it may open a way of the clear analysis for various aperiodic systems. Once the MO of the giant molecule model is obtained, to analyze the

band dispersion, the energy levels should be classified with the crystalline momentum.

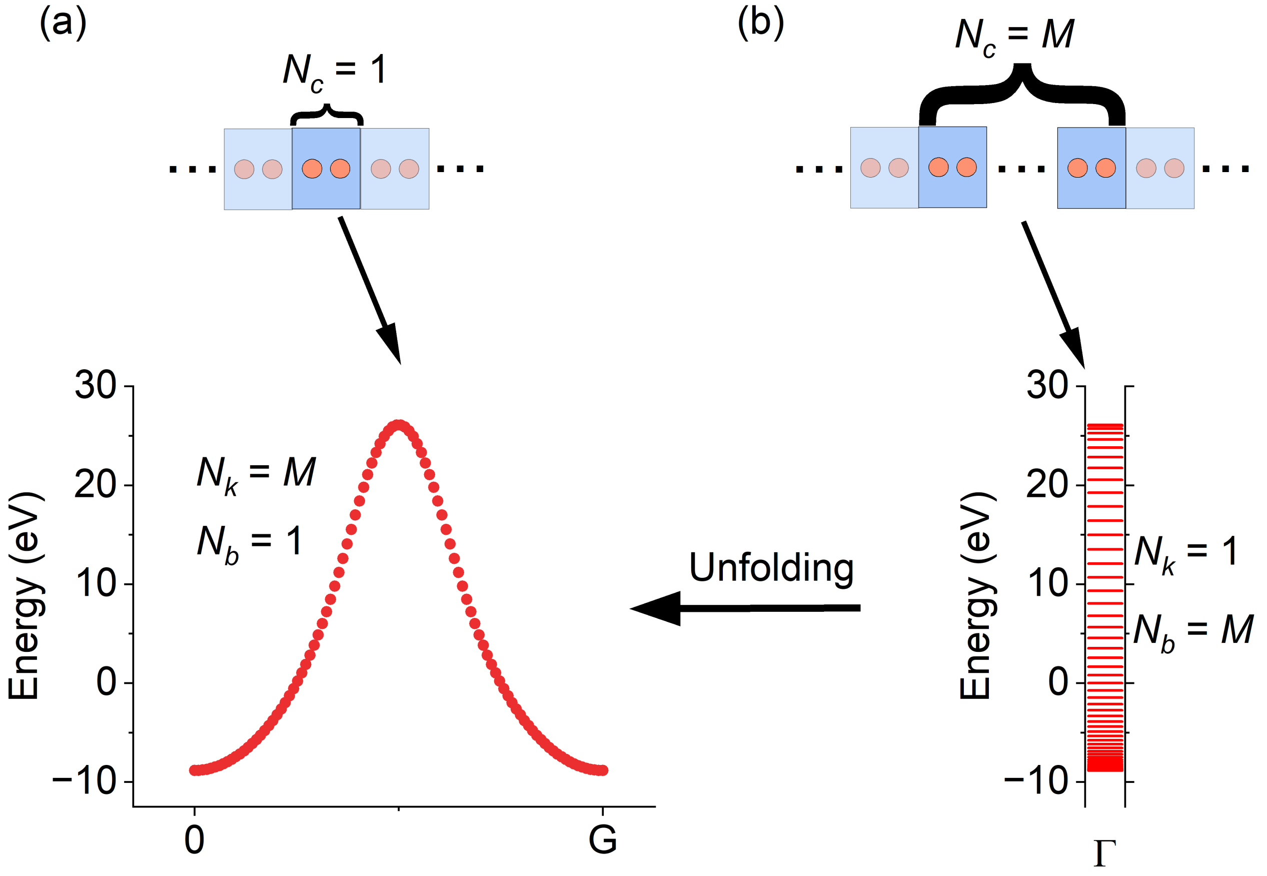

Figure S1: Schematic of extraction of the band dispersion described in the FBZ for a primitive cell from energy levels in a supercell: (a) the band dispersion in an infinite chain modeled by a primitive cell with two atoms; (b) the energy levels at the Γ-point in an infinite chain modeled by a supercell of N_c cells, where N_k and N_b are the number of sampled k-points and bands, respectively, and G is the period of the reciprocal lattice for the primitive cell, and it is assumed that the two atoms in a cell are hydrogen atoms, and that there is spin degeneracy, for simplicity.

Here, we propose the GMBU procedure to apply the band unfolding method to the classification to get the spectral weights for the band dispersion from the MO energy levels of the giant molecule model. The unfolding method is a technique that extract the band dispersion from the electronic structures for an ideal supercell and represent the spectral weight in the reference FBZ[1–10]. Let us consider a simple model of a hydrogen chain. First, we consider an infinite chain model. We can set the unit cell for the simulation to a primitive cell including two atoms as shown in Fig. S1(a). The band dispersion can be evaluated with

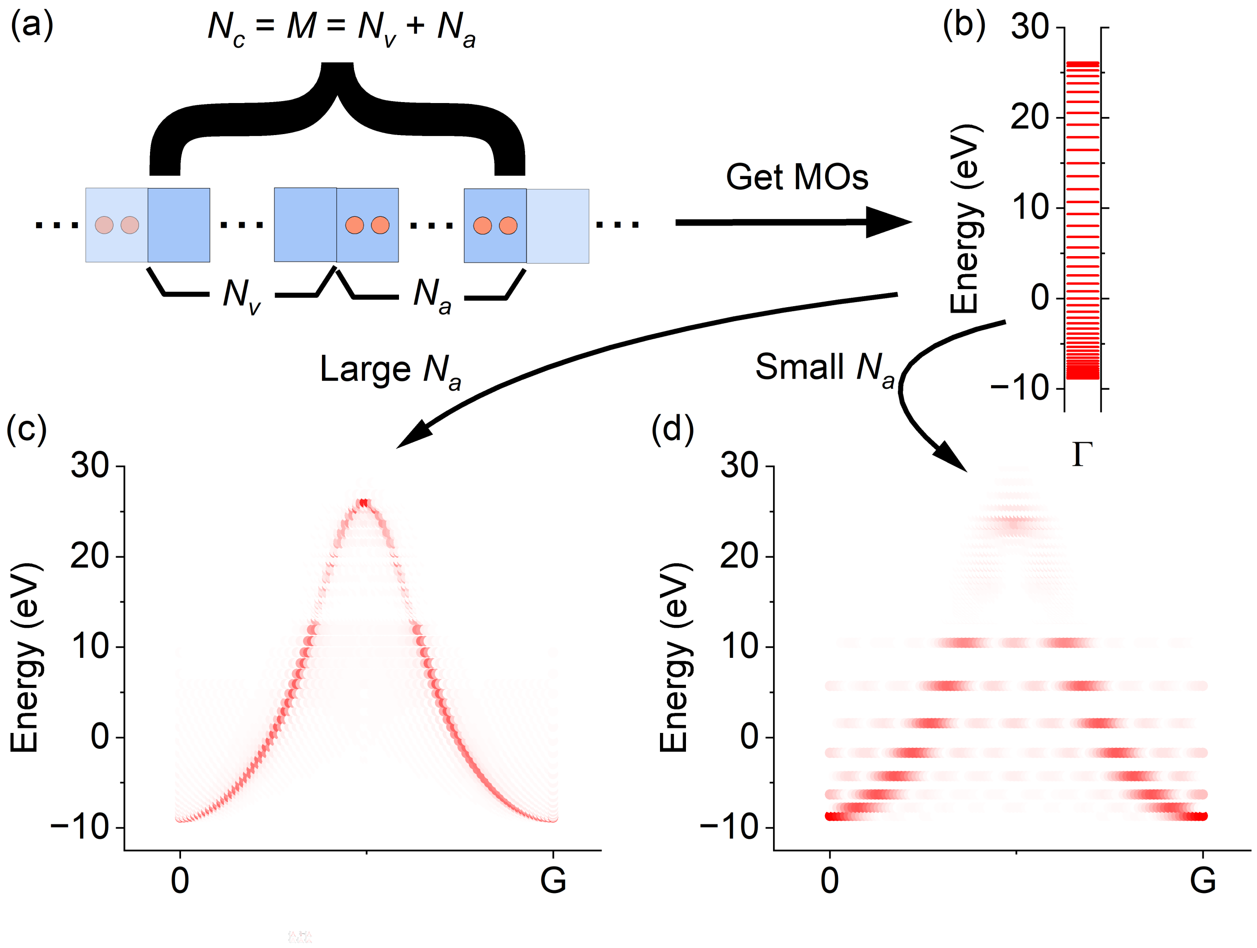

Figure S2: Schematic of extraction of the band dispersion described in the FBZ for a primitive cell from MO energy levels of a finite chain molecule: (a) a supercell model of a finite chain; (b) the energy levels at the Γ-point in a finite chain modeled by a supercell consisting of N_v vacant cells and N_a cells including atoms; (c, d) the band dispersion in a finite chain unfolded from the MO energy levels in the case of (c) large and (d) small N_a, where G is the period of the reciprocal lattice for the primitive cell.

the number of sampled k-points $N_k = M$. We can also consider the case that the unit cell is M times as large as the previous unit cell and each interatomic distance is fixed, and the M energy levels at the Γ-point are given as shown in Fig. S1(b). Here, we can retrieve the band dispersion for the primitive cell case by unfolding the energy levels at the Γ-point for the supercell case. In replacing several cells with vacant cells, the system is changed to a finite chain, that is, one-dimensional molecule, and the energy levels can be obtained in the same way (Fig. S2(a, b)). In addition, the band unfolding can also be done, because for the supercell, we can introduce vacancies or impurities, where it is possible to apply the band unfolding method. Indeed, in removing an atom from the supercell, the unfolded band dispersion is slightly influenced by the perturbation owing to the vacancy (Fig. S2(c)). It is also possible to introduce more continuous vacancies while the reminder of atoms is connected with one another and to perform the band unfolding calculations formally. In this case, the unfolded band dispersion can be kept for a finite chain with enough atoms (Fig. S2(c)), and it changes from the continuous picture of the dispersive bands to the discrete picture of the MOs in terms of the energy axis as the number of atoms in the center domain decreases (Fig. S2(d)). To review the above, since the models that the continuous vacancies are introduced describe finite chains, as a result, this procedure can be applied to extraction of the band dispersion in finite or aperiodic systems. For two- and three-dimensional cases, the finite systems are expressed by flakes and molecules, respectively, and how to extract the band dispersion in finite systems can be demonstrated in the same way as the one-dimensional chain case.

Through a linear-combination-of-atomic-orbital (LCAO) unfolding method[6], we describe how to get the spectral weights of a finite system. First, we will outline how unfolding is performed in a system with periodicity. The spectral function operator is defined as $\hat{A}(\epsilon) = -(1/\pi)\mathrm{Im}\hat{G}(\epsilon + \mathrm{i}\eta)$, where i is the imaginary unit, η is the positive infinitesimal, $\hat{G}(z) = \sum_{KJ} |\Psi_J^{(\boldsymbol{K})}\rangle \langle\Psi_J^{(\boldsymbol{K})}| /(z - \epsilon_{\boldsymbol{K}J})$, and $|\Psi_J^{(\boldsymbol{K})}\rangle$ is the Bloch wavefunction for the J-th energy eigenvalue $\epsilon_{\boldsymbol{K}J}$ at the k-point with wave vector $\boldsymbol{K}$. For simplicity, first we explain the

cases that spin polarization is not considered. We expand the Bloch wavefunction with the AOs: $\Psi_J^{(\boldsymbol{K})}(\mathbf{r}) = (1/\sqrt{L})\sum_{\boldsymbol{R}} e^{\mathrm{i}\boldsymbol{K}\cdot\boldsymbol{R}} \sum_N c_{NJ}^{(\boldsymbol{K})} \phi_N^{\boldsymbol{R}}(\mathbf{r})$, where $\mathbf{r}$ is the position, L is the number of cells under the Born–von Karman boundary condition, $\boldsymbol{R}$ is a lattice vector, N is an AO index, $c_{NJ}^{(\boldsymbol{K})}$ is an expansion coefficient of the N-th AO for the Bloch wavefunction of state J, and $\phi_N^{\boldsymbol{R}}$ is an AO in a cell moved by $\boldsymbol{R}$ from the original unit cell. Each AO corresponds to an orbital belonging to an atom. In the band unfolding, reference cells for projection of the band dispersion are introduced, and lower-case variables such as $\boldsymbol{k}$, j, $\boldsymbol{r}$ are the counterparts for the reference cells. We get spectral function (see also section 6):

$$\bar{A}(\boldsymbol{k},\epsilon) = \sum_j \left\langle \psi_j^{(\boldsymbol{k})} \middle| \hat{A}(\epsilon) \middle| \psi_j^{(\boldsymbol{k})} \right\rangle$$

$$= \gamma \sum_{\boldsymbol{K}J\boldsymbol{G}} \delta_{\boldsymbol{k}-\boldsymbol{G},\boldsymbol{K}} \sum_{MN\boldsymbol{r}'} e^{\mathrm{i}\boldsymbol{k}\cdot(\boldsymbol{r}'-\boldsymbol{r}_0(N))} c_{MJ}^{(\boldsymbol{K})*} c_{NJ}^{(\boldsymbol{K})} \left\langle \phi_M^{\boldsymbol{0}} \middle| \phi_{n(N)}^{\boldsymbol{r}'} \right\rangle \delta(\epsilon - \epsilon_{\boldsymbol{K}J})$$

$$= \gamma \sum_J \sum_{MN\boldsymbol{r}'} e^{\mathrm{i}\boldsymbol{k}\cdot(\boldsymbol{r}'-\boldsymbol{r}_0(N))} c_{MJ}^{(\boldsymbol{K}')*} c_{NJ}^{(\boldsymbol{K}')} \left\langle \phi_M^{\boldsymbol{0}} \middle| \phi_{n(N)}^{\boldsymbol{r}'} \right\rangle \delta(\epsilon - \epsilon_{\boldsymbol{K}'J}). \tag{1}$$

Note that $\psi_j^{(\boldsymbol{k})}$ is one of the Bloch wavefunctions in the reference cell, that we give $\boldsymbol{K}'$ equal to $\boldsymbol{K}$ so that $\boldsymbol{k} - \boldsymbol{G}' = \boldsymbol{K}$, where $\boldsymbol{G}'$ is also a reciprocal lattice vector, and that $n(N)$ stands for an AO index in the reference cell that the N-th PAO in the original unit cell mapped to, $\delta(\epsilon - \epsilon_{\boldsymbol{K}'J})$ is a delta function, $\boldsymbol{G}$ is a reciprocal lattice vector, and γ is the ratio of the number of cells L/l. Assuming that all the atoms are located at sites forming periodical lattices, we also get

$$\sum_{\boldsymbol{r}'} e^{\mathrm{i}\boldsymbol{k}\cdot(\boldsymbol{r}'-\boldsymbol{r}_0(N))} \left\langle \phi_M^{\boldsymbol{0}} \middle| \phi_{n(N)}^{\boldsymbol{r}'} \right\rangle = \sum_{N'\boldsymbol{R}} \delta_{n(N),n'(N')} e^{\mathrm{i}\boldsymbol{k}\cdot(\boldsymbol{R}+\boldsymbol{r}_0(N')-\boldsymbol{r}_0(N))} \left\langle \phi_M^{\boldsymbol{0}} \middle| \phi_{N'}^{\boldsymbol{R}} \right\rangle \equiv \mathcal{S}_{MN}^{(\boldsymbol{k})}, \tag{2}$$

where $\delta_{n(N),n(N')}$ is the Kronecker delta, and we defined the mapping matrix as $\mathcal{S}^{((k))}$. From Eqs. 1 and 2, we get spectral weight $\mathcal{A}_{\boldsymbol{k}J} = \bar{A}(\boldsymbol{k},\epsilon_{\boldsymbol{K}',J})/\delta(0)$ as follows, except for the

degenerate points about $\epsilon_{\boldsymbol{K}',J}$.

$$\mathcal{A}_{\boldsymbol{k}J} \equiv \gamma \sum_{MN} c_{MJ}^{(\boldsymbol{K}')*} c_{NJ}^{(\boldsymbol{K}')} \sum_{N'\boldsymbol{R}} \delta_{n(N),n(N')} e^{\mathrm{i}\boldsymbol{k}\cdot(\boldsymbol{R}+\boldsymbol{r}_0(N')-\boldsymbol{r}_0(N))} \left\langle \phi_M^{\boldsymbol{0}} \,\middle|\, \phi_{N'}^{\boldsymbol{R}} \right\rangle = \gamma \left(c^{(\boldsymbol{K}')\dagger} \mathcal{S}^{(\boldsymbol{k})} c^{(\boldsymbol{K}')} \right)_{JJ}. \tag{3}$$

Note that the above definition of the spectral weight can be diverted to the case with degeneracy and is equivalent with the definition in the previous study of Lee et al.[6]. Furthermore, the phase shift can be expressed with atomic coordinates instead of lattice vectors:

$$\mathcal{A}_{\boldsymbol{k}J} \simeq \gamma \sum_{MN} c_{MJ}^{(\boldsymbol{K}')*} c_{NJ}^{(\boldsymbol{K}')} \sum_{N'\boldsymbol{R}} \delta_{n(N),n(N')} e^{\mathrm{i}\boldsymbol{k}\cdot(\boldsymbol{R}+\tau(N')-\tau(N))} \left\langle \phi_M^{\boldsymbol{0}} \,\middle|\, \phi_{N'}^{\boldsymbol{R}} \right\rangle, \tag{4}$$

where $\tau(N)$ is the position of an atom that the N-th AO belongs. Once $c^{(\boldsymbol{K}')}$ is obtained from the eigenvalue problem, the computational cost to evaluate $\left(c^{(\boldsymbol{K}')\dagger} \mathcal{S}^{(\boldsymbol{k})} c^{(\boldsymbol{K}')} \right)_{JJ}$ is $O(\mathrm{N}^2\mathrm{nM})$, where N is the total number of AOs, n is the number of states within an energy range to focus on, and M is the number of k-points along a k-path that the band dispersion is investigated. The overall computational cost is mainly $O(\mathrm{N}^3\mathrm{M})$ coming from the (generalized) eigenvalue problem because of $\mathrm{n} \ll \mathrm{N}$. In the same way, it is possible to extend the above formulation to cases with spin polarization. Especially in the case that the basis set is expressed with two-component spinors, $\mathcal{A}_{\boldsymbol{k}J} = \gamma \left(c^{(\boldsymbol{K}')\dagger} \left(\sigma_0 \otimes \mathcal{S}^{(\boldsymbol{k})} \right) c^{(\boldsymbol{K}')} \right)_{JJ}$, where σ_0 is the 2×2 identity matrix. For a finite system, the primitive cell of the reciprocal lattice can include only $\boldsymbol{K} = \boldsymbol{0}$ (i.e. the Γ-point), and therefore, in the GMBU procedure, $\mathcal{A}_{\boldsymbol{k}J} = \gamma \left(c^{(\boldsymbol{0})\dagger} \mathcal{S}^{(\boldsymbol{k})} c^{(\boldsymbol{0})} \right)_{JJ}$: only mapping matrix $\mathcal{S}^{(\boldsymbol{k})}$ depends on the crystalline momentum, and $\mathcal{S}^{(\boldsymbol{k})}$ projects the spectral weight on the band dispersion relation. The overall computational cost reduces to $O(\mathrm{N}^2\mathrm{nM})$ in the case of $O(\mathrm{nM}) > O(\mathrm{N})$ because the eigenvalue problem should be solved only at $\boldsymbol{K} = \boldsymbol{0}$. In the case of the distorted system with ambiguous cells, instead of the ratio of the number of cells, the ratio of the number of atoms could be set to γ. As the system size is larger, MO energy levels $\epsilon_{\boldsymbol{0}J}$ become distributed more broadly along the energy axis. Therefore, finite $\mathcal{A}_{\boldsymbol{k}J}$ can appear on such dense energy levels that the almost continuous energy band can be formed. This picture is consistent with formation of energy bands in the condensed matter.

In short, the GMBU procedure evaluates the k-dependent mapping matrices to extract the band dispersion relation in addition to the band widths described by the k-independent MO energy levels.

2. Computational model of a 15×15 graphene nanoflake model

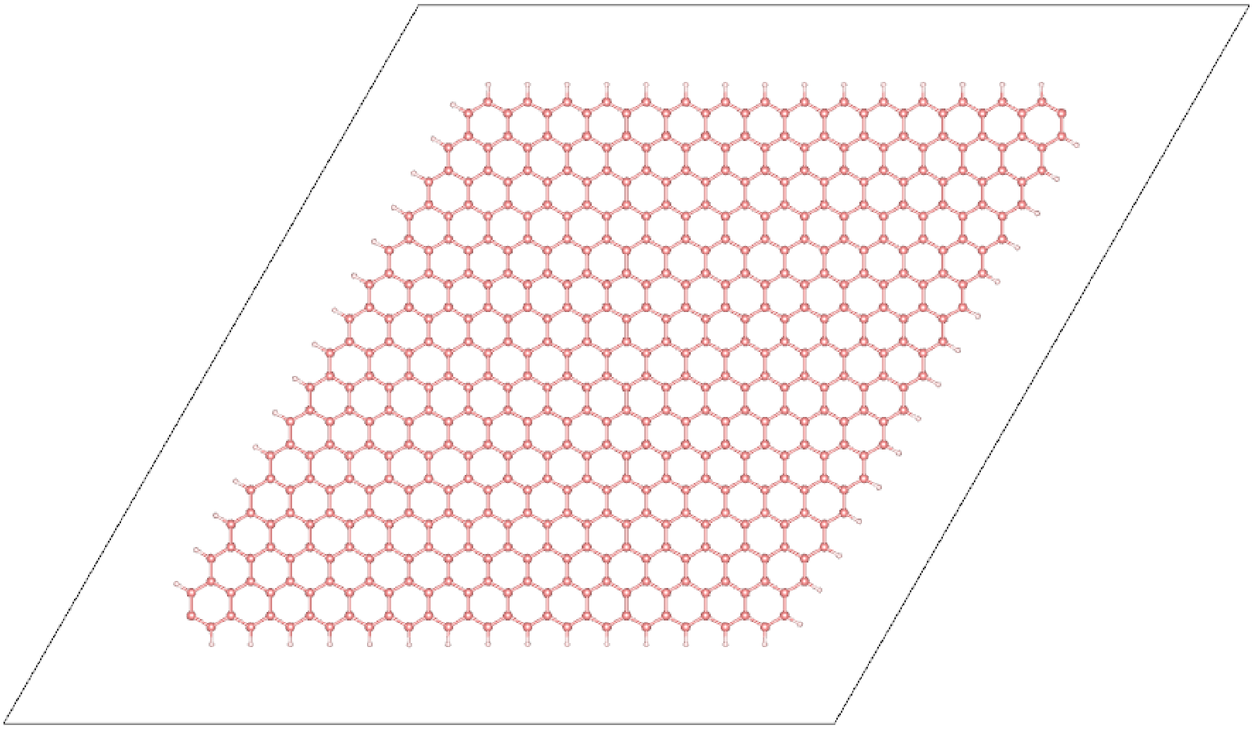

Figure S3: Top view of a graphene nanoflake model of 15×15 rings. The dark brown and light brown balls represent carbon and hydrogen atoms, respectively, and sticks stand for bonds between atoms. The rhombus represents the unit cell.

We used a 15×15 graphene nanoflake model shown in Fig. S3. After expanding the unit cell and creating the flake model, only the positions of hydrogen atoms were optimized using the same computational conditions.

3. Computational model of a 9×9 tungsten disulfide nanoflake model

We used a 9×9 WS_2 nanoflake model. First, to build the 9×9 WS_2 flake model, we optimized the lattice constants and atomic positions in the bulk WS_2 system. After structural optimization, the in-plane lattice constant of WS_2 (a_{WS_2}) was 3.20 Å and the distance between the tungsten and sulfur atoms (d_{W-S}) was 2.44 Å, which is in good agreement with values from the previous study[11] ($a_{WS_2} = 3.19$ Å; $d_{W-S} = 2.42$ Å). Then the unit cell was expanded and flakes were modeled. After the flake was modeled, no geometry optimization was considered.

4. Computational model of a 7×7 Bi/Ag(111)-$(\sqrt{3} \times \sqrt{3})R30^\circ$ surface alloy nanoflake model

As shown in Fig. S4 and Fig. S5, we constructed a 7×7 Bi/Ag(111)-$(\sqrt{3} \times \sqrt{3})R30^\circ$ surface alloy nanoflake model based on the previous study[12]. We considered only one monolayer of the alloyed system on the surface of Ag(111) and constructed the lattice of the surface alloy based on the experimental lattice constant of silver, and the in-plane lattice constant was 5.0215 Å. In the surface alloy system, the bismuth atoms cause corrugation due to the size of their atomic radius, and the corrugation parameter, i.e., the distance between the plane formed by the bismuth atoms and the plane formed by the silver atoms, was set to 0.69 Å. This corrugation parameter was given by a calculated value from the optimization in a two-atomic layer model in the previous study. After the flake was modeled, no geometry

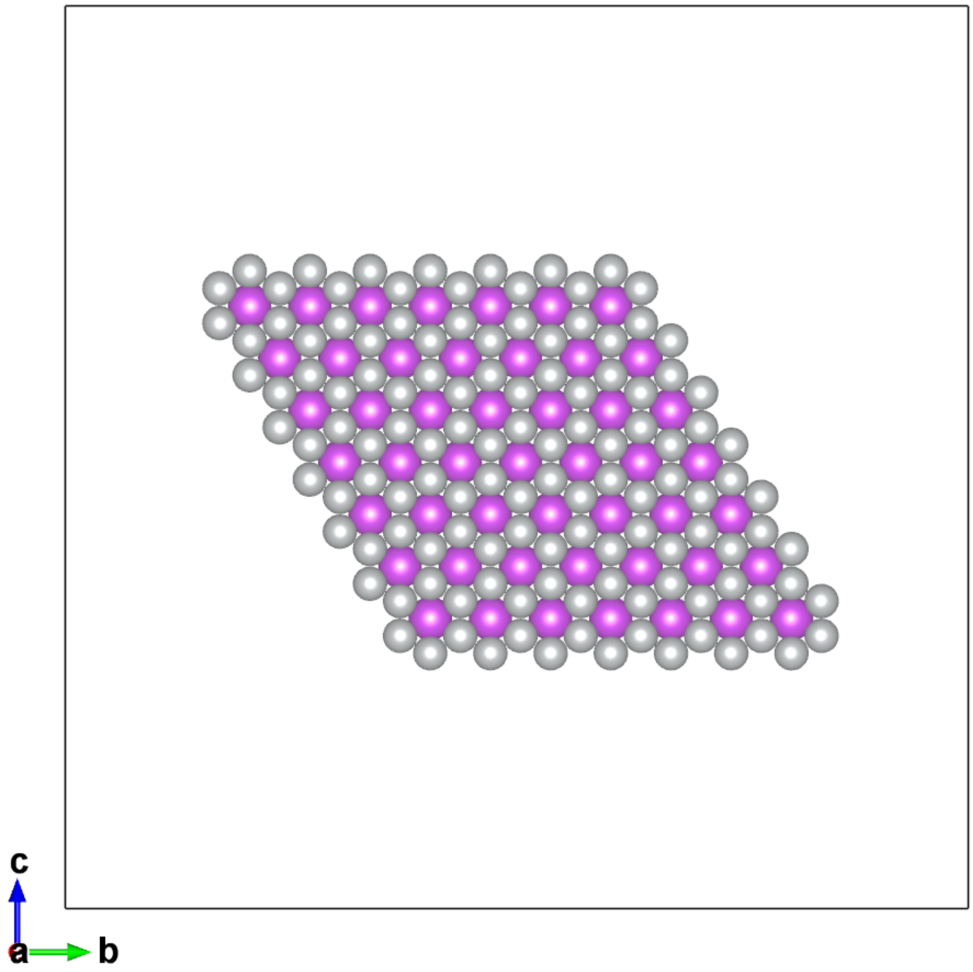

Figure S4: Top view of a Bi/Ag(111)-($\sqrt{3} \times \sqrt{3})R30^{\circ}$ nanoflake model of 7×7 rings. The gray and purple balls represent silver and bismuth atoms, respectively. The square represents the unit cell.

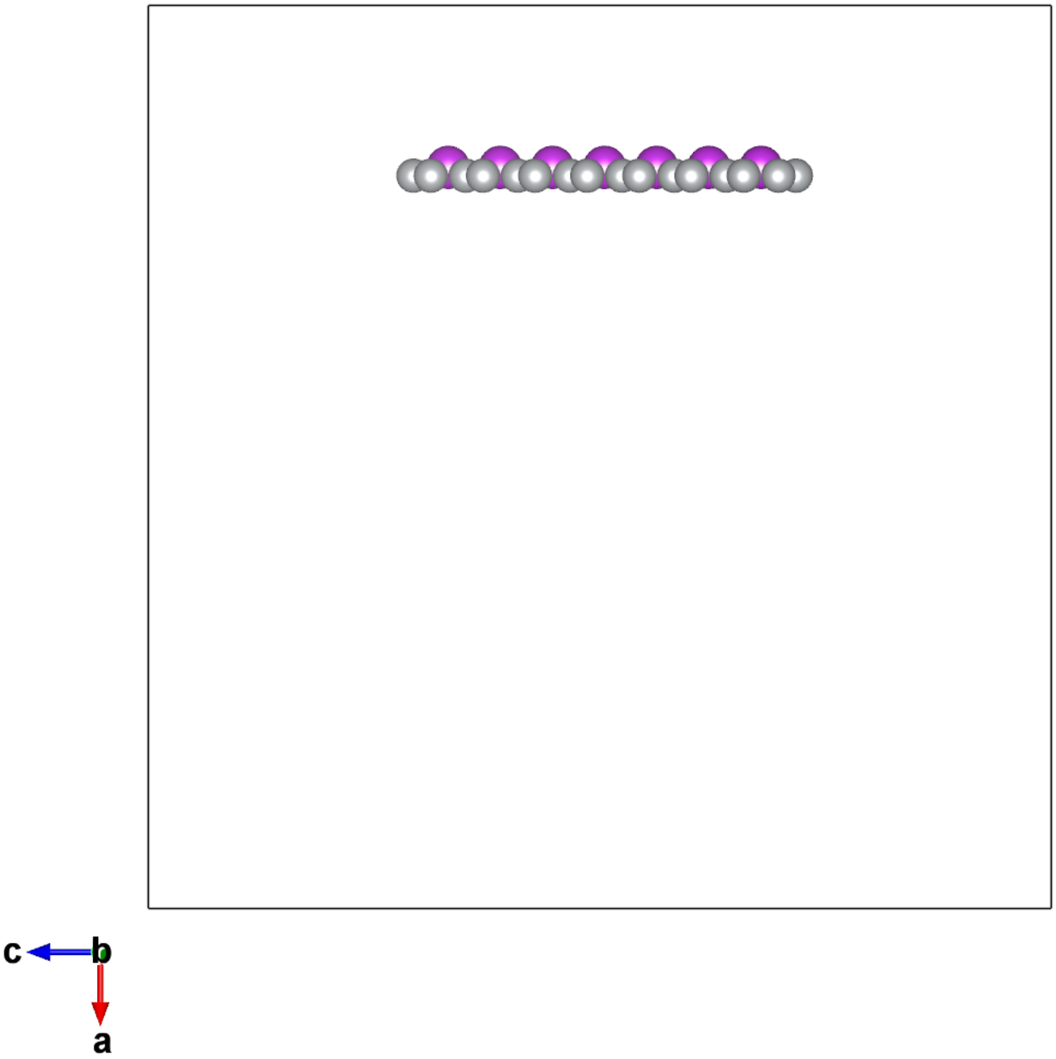

Figure S5: Side view of a Bi/Ag(111)-($\sqrt{3} \times \sqrt{3})R30^\circ$ nanoflake model of 7×7 rings. The gray and purple balls represent silver and bismuth atoms, respectively. The black square represents the unit cell.

optimization was considered. Since the surface states that yield spin splitting are strongly localized to a single layer of the surface, modeling with monoatomic layers is valid[12,13].

5. Band dispersion extracted from a 7×7 Bi/Ag(111)-$(\sqrt{3} \times \sqrt{3})R30^\circ$ surface alloy nanoflake model

To illustrate the application of the in-plane spin components as well, we also applied the GMBU procedure to a Bi/Ag(111)-$(\sqrt{3}\times\sqrt{3})R30^\circ$ surface alloy nanoflake. The bismuth/silver surface alloy exhibits giant Rashba spin splitting[14], which is caused by the spin-orbit interaction and spatial inversion symmetry breaking along the out-of-plane direction. The Rashba spin splitting is a spin splitting where the spin direction is in the in-plane direction. The band dispersion images for spin x polarization and spin y polarization in the flakes in Fig. S6(a) and Fig. S6(c) are in good agreement with those in the periodic perfect system in Figures Fig. S6(b) and Fig. S6(d), respectively. A large Rashba spin splitting around the Γ-point near the Fermi level was also confirmed even in the case of a flake. This is supported by the fact that the distribution of the positive and negative signs of the spin direction in the band dispersion images of the x- and y-polarization components match.

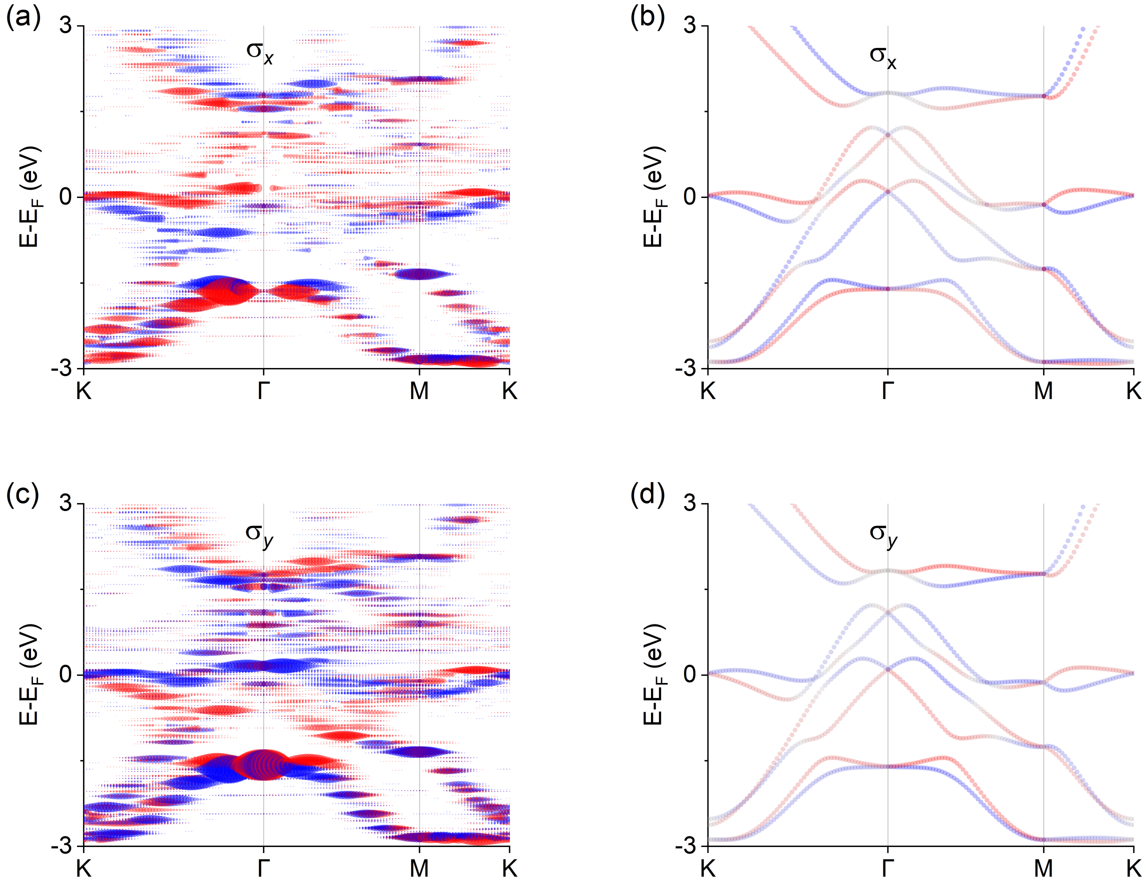

Figure S6: (a, c) Spin-resolved band dispersion extracted from a Bi/Ag(111)-$(\sqrt{3} \times \sqrt{3})R30°$ surface alloy flake model of 7×7 rings. The k-path connects special k-points of Γ(0, 0, 0), M($(2\pi/a_{\mathrm{Ag}(111)})(1/2, 0, 0)$), and K($(2\pi/a_{\mathrm{Ag}(111)})(1/3, 1/3, 0)$), where $a_{\mathrm{Ag}(111)}$ is the lattice constant of a pristine Ag(111) surface. The blue and red circles represent the negative and positive signs of (a) normalized spin-x-polarized spectral weights $\langle\sigma_x\rangle_{\boldsymbol{k}J}/\mathcal{A}_{\boldsymbol{k}J}$ or (c) normalized spin-y-polarized spectral weights $\langle\sigma_y\rangle_{\boldsymbol{k}J}/\mathcal{A}_{\boldsymbol{k}J}$, respectively, and the radius of each circle reflects the magnitude of $\mathcal{A}_{\boldsymbol{k}J}$. The points that $\mathcal{A}_{\boldsymbol{k}J}$ less than 0.01 are omitted. (b, d) Spin-resolved band dispersion with respect to (b) $\langle\sigma_x\rangle_{\boldsymbol{k}J}$ or (d) $\langle\sigma_y\rangle_{\boldsymbol{k}J}$ of a pristine Bi/Ag(111)-$(\sqrt{3} \times \sqrt{3})R30°$ surface alloy as a comparison with (a, c). The Fermi level is set to the origin of the energy axis.

6. Derivation of spectral weight represented by reference-cell Bloch functions

The spectral function operator is defined as $\hat{A}(\epsilon) = -(1/\pi)\mathrm{Im}\hat{G}(\epsilon + \mathrm{i}\eta)$, where i is the imaginary unit, η is the positive infinitesimal, $\hat{G}(z) = \sum_{KJ} \left|\Psi_J^{(\boldsymbol{K})}\right\rangle \left\langle\Psi_J^{(\boldsymbol{K})}\right| / (z - \epsilon_{\boldsymbol{K}J})$, and $\Psi_J^{(\boldsymbol{K})}$ is the Bloch wavefunction for the J-th energy eigenvalue $\epsilon_{\boldsymbol{K}J}$ at the k-point with wave vector $\boldsymbol{K}$. For simplicity, we explain the cases that spin polarization is not considered. Each AO corresponds to an orbital belonging to an atom. In the band unfolding, reference cells for projection of the band dispersion are introduced, and here, lower-case variables such as $\boldsymbol{k}$, j stand for the counterparts for the reference cells. Represented by the Bloch wavefunctions of the reference cell $\psi_j^{(\boldsymbol{k})}$, the spectral function can be given as

$$\bar{A}(\boldsymbol{k}, \epsilon) = \sum_j \left\langle \psi_j^{(\boldsymbol{k})} \middle| \hat{A}(\epsilon) \middle| \psi_j^{(\boldsymbol{k})} \right\rangle, \tag{5}$$

where

$$\left\langle \psi_j^{(\boldsymbol{k})} \middle| \hat{A}(\epsilon) \middle| \psi_j^{(\boldsymbol{k})} \right\rangle = \sum_{\boldsymbol{K}J} \left| \left\langle \psi_j^{(\boldsymbol{k})} \middle| \Psi_J^{(\boldsymbol{K})} \right\rangle \right|^2 \left\langle \Psi_J^{(\boldsymbol{K})} \middle| \hat{A}(\epsilon) \middle| \Psi_J^{(\boldsymbol{K})} \right\rangle = \sum_{\boldsymbol{K}J} \left| \left\langle \psi_j^{(\boldsymbol{k})} \middle| \Psi_J^{(\boldsymbol{K})} \right\rangle \right|^2 \delta(\epsilon - \epsilon_{\boldsymbol{K}J}),$$

$$\left\langle \psi_j^{(\boldsymbol{k})} \middle| \Psi_J^{(\boldsymbol{K})} \right\rangle = \sum_{mN} c_{mj}^{(\boldsymbol{k})*} \left\langle \varphi_m^{(\boldsymbol{k})} \middle| \Phi_N^{(\boldsymbol{K})} \right\rangle c_{NJ}^{(\boldsymbol{K})}, \tag{6}$$

$$\left|\Psi_J^{(\boldsymbol{K})}\right\rangle = \sum_N \left|\Phi_N^{(\boldsymbol{K})}\right\rangle c_{NJ}^{(\boldsymbol{K})}, \tag{7}$$

$$\left|\psi_j^{(\boldsymbol{k})}\right\rangle = \sum_n \left|\varphi_n^{(\boldsymbol{k})}\right\rangle c_{nj}^{(\boldsymbol{k})}, \tag{8}$$

$$\left|\Phi_N^{(\boldsymbol{K})}\right\rangle = \frac{1}{\sqrt{L}} \sum_{\boldsymbol{R}} \left|\phi_N^{\boldsymbol{R}}\right\rangle e^{\mathrm{i}\boldsymbol{K}\cdot\boldsymbol{R}}, \tag{9}$$

$$\left|\varphi_n^{(\boldsymbol{k})}\right\rangle = \frac{1}{\sqrt{l}} \sum_{\boldsymbol{r}} \left|\phi_n^{\boldsymbol{r}}\right\rangle e^{\mathrm{i}\boldsymbol{k}\cdot\boldsymbol{r}}, \tag{10}$$

$\delta(\epsilon - \epsilon_{\boldsymbol{K}J})$ is a delta function, L is the number of cells under the Born–von Karman boundary condition, $\boldsymbol{R}$ is a lattice vector, N is an AO index, $c_{NJ}^{(\boldsymbol{K})}$ is an expansion coefficient of the

N-th AO for the Bloch wavefunction of state J, and $\phi_N^{\boldsymbol{R}}$ is an AO in a cell moved by $\boldsymbol{R}$ from the original unit cell. Then,

$$\sum_j \left|\left\langle \psi_j^{(\boldsymbol{k})} \middle| \Psi_J^{(\boldsymbol{K})} \right\rangle\right|^2 = \sum_{MnjmN} c_{MJ}^{(\boldsymbol{K})*} \left\langle \mathit{\Phi}_M^{(\boldsymbol{K})} \middle| \varphi_n^{(\boldsymbol{k})} \right\rangle c_{nj}^{(\boldsymbol{k})} c_{mj}^{(\boldsymbol{k})*} \left\langle \varphi_m^{(\boldsymbol{k})} \middle| \mathit{\Phi}_N^{(\boldsymbol{K})} \right\rangle c_{NJ}^{(\boldsymbol{K})}, \tag{11}$$

$$\left\langle \varphi_m^{(\boldsymbol{k})} \middle| \mathit{\Phi}_N^{(\boldsymbol{K})} \right\rangle = \frac{1}{\sqrt{lL}} \sum_{\boldsymbol{rR}} \left\langle \phi_m^{\boldsymbol{r}} \middle| \phi_N^{\boldsymbol{R}} \right\rangle e^{\mathrm{i}(\boldsymbol{K}\cdot\boldsymbol{R}-\boldsymbol{k}\cdot\boldsymbol{r})} = \frac{1}{\sqrt{lL}} \sum_{\boldsymbol{rR}} \left\langle \phi_m^{\boldsymbol{r}} \middle| \phi_N^{\boldsymbol{R}} \right\rangle e^{\mathrm{i}(\boldsymbol{K}-\boldsymbol{k})\cdot\boldsymbol{R}} e^{\mathrm{i}\boldsymbol{k}\cdot(\boldsymbol{R}-\boldsymbol{r})}$$

$$= \frac{1}{\sqrt{lL}} \sum_{\boldsymbol{R}} e^{\mathrm{i}(\boldsymbol{K}-\boldsymbol{k})\cdot\boldsymbol{R}} \sum_{\boldsymbol{r}} \left\langle \phi_m^{\boldsymbol{r}} \middle| \phi_{n'(N)}^{\boldsymbol{R}+\boldsymbol{r}_0(N)} \right\rangle e^{\mathrm{i}\boldsymbol{k}\cdot(\boldsymbol{R}-\boldsymbol{r})}$$

$$= \frac{1}{\sqrt{lL}} e^{-\mathrm{i}\boldsymbol{k}\cdot\boldsymbol{r}_0(N)} \sum_{\boldsymbol{R}} e^{\mathrm{i}(\boldsymbol{K}-\boldsymbol{k})\cdot\boldsymbol{R}} \sum_{\boldsymbol{r}'} \left\langle \phi_m^{\boldsymbol{0}} \middle| \phi_{n'(N)}^{\boldsymbol{r}'} \right\rangle e^{\mathrm{i}\boldsymbol{k}\cdot\boldsymbol{r}'} = \sqrt{\frac{L}{l}} e^{-\mathrm{i}\boldsymbol{k}\cdot\boldsymbol{r}_0(N)} \sum_{\boldsymbol{G}} \delta_{\boldsymbol{k}-\boldsymbol{G},\boldsymbol{K}} \left\langle \varphi_m^{(\boldsymbol{k})} \middle| \varphi_{n'(N)}^{(\boldsymbol{k})} \right\rangle. \tag{12}$$

$$\sum_{jm} c_{nj}^{(\boldsymbol{k})} c_{mj}^{(\boldsymbol{k})*} \left\langle \varphi_m^{(\boldsymbol{k})} \middle| \mathit{\Phi}_N^{(\boldsymbol{K})} \right\rangle = \sum_m \left\langle \tilde{\varphi}_n^{(\boldsymbol{k})} \middle| \tilde{\varphi}_m^{(\boldsymbol{k})} \right\rangle \left\langle \varphi_m^{(\boldsymbol{k})} \middle| \mathit{\Phi}_N^{(\boldsymbol{K})} \right\rangle$$

$$= \sqrt{\frac{L}{l}} e^{-\mathrm{i}\boldsymbol{k}\cdot\boldsymbol{r}_0(N)} \sum_{\boldsymbol{G}} \delta_{\boldsymbol{k}-\boldsymbol{G},\boldsymbol{K}} \sum_m \left\langle \tilde{\varphi}_n^{(\boldsymbol{k})} \middle| \tilde{\varphi}_m^{(\boldsymbol{k})} \right\rangle \left\langle \varphi_m^{(\boldsymbol{k})} \middle| \varphi_{n'(N)}^{(\boldsymbol{k})} \right\rangle = \sqrt{\frac{L}{l}} e^{-\mathrm{i}\boldsymbol{k}\cdot\boldsymbol{r}_0(N)} \sum_{\boldsymbol{G}} \delta_{\boldsymbol{k}-\boldsymbol{G},\boldsymbol{K}} \delta_{nn'(N)}, \tag{13}$$

where $n'(N)$ is the function mapping N to n', $\tilde{\varphi}^{(\boldsymbol{k})}$ is the dual orbital for $\varphi^{(\boldsymbol{k})}$, and $\delta_{\boldsymbol{k}-\boldsymbol{G},\boldsymbol{K}}$ is the Kronecker delta. Therefore, Eq. 11 can be rewritten as

$$\sum_j \left|\left\langle \psi_j^{(\boldsymbol{k})} \middle| \Psi_J^{(\boldsymbol{K})} \right\rangle\right|^2 = \sqrt{\frac{L}{l}} e^{-\mathrm{i}\boldsymbol{k}\cdot\boldsymbol{r}_0(N)} \sum_{\boldsymbol{G}} \delta_{\boldsymbol{k}-\boldsymbol{G},\boldsymbol{K}} \sum_{MN} c_{MJ}^{(\boldsymbol{K})*} \left\langle \mathit{\Phi}_M^{(\boldsymbol{K})} \middle| \varphi_{n'(N)}^{(\boldsymbol{k})} \right\rangle c_{NJ}^{(\boldsymbol{K})}. \tag{14}$$

Then,

$$\left\langle \mathit{\Phi}_M^{(\boldsymbol{K})} \middle| \varphi_{n'(N)}^{(\boldsymbol{k})} \right\rangle = \frac{1}{\sqrt{Ll}} \sum_{\boldsymbol{Rr}} \left\langle \phi_M^{\boldsymbol{R}} \middle| \phi_{n'(N)}^{\boldsymbol{r}} \right\rangle e^{\mathrm{i}(\boldsymbol{k}\cdot\boldsymbol{r}-\boldsymbol{K}\cdot\boldsymbol{R})}$$

$$= \frac{1}{\sqrt{Ll}} \sum_{\boldsymbol{Rr}} \left\langle \phi_M^{\boldsymbol{R}} \middle| \phi_N^{\boldsymbol{r}} \right\rangle e^{\mathrm{i}(\boldsymbol{k}-\boldsymbol{K})\cdot\boldsymbol{R}} e^{\mathrm{i}\boldsymbol{k}\cdot(\boldsymbol{r}-\boldsymbol{R})} = \frac{1}{\sqrt{Ll}} \sum_{\boldsymbol{R}} e^{\mathrm{i}(\boldsymbol{k}-\boldsymbol{K})\cdot\boldsymbol{R}} \sum_{\boldsymbol{r}'} \left\langle \phi_M^{\boldsymbol{0}} \middle| \phi_{n'(N)}^{\boldsymbol{r}'} \right\rangle e^{\mathrm{i}\boldsymbol{k}\cdot\boldsymbol{r}'}$$

$$= \sqrt{\frac{L}{l}} \sum_{\boldsymbol{G}} \delta_{\boldsymbol{k}-\boldsymbol{G},\boldsymbol{K}} \sum_{\boldsymbol{r}'} \left\langle \phi_M^{\boldsymbol{0}} \middle| \phi_{n'(N)}^{\boldsymbol{r}'} \right\rangle e^{\mathrm{i}\boldsymbol{k}\cdot\boldsymbol{r}'}. \tag{15}$$

Therefore,

$$\sum_{j}\left|\left\langle\psi_j^{(\boldsymbol{k})}\,\middle|\,\Psi_J^{(\boldsymbol{K})}\right\rangle\right|^2=\frac{L}{l}\sum_{\boldsymbol{G}}\delta_{\boldsymbol{k}-\boldsymbol{G},\boldsymbol{K}}\sum_{MN\boldsymbol{r}'}e^{\mathrm{i}\boldsymbol{k}\cdot(\boldsymbol{r}'-\boldsymbol{r}_0(N))}c_{MJ}^{(\boldsymbol{K})*}c_{NJ}^{(\boldsymbol{K})}\left\langle\phi_M^{\boldsymbol{0}}\,\middle|\,\phi_{n'(N)}^{\boldsymbol{r}'}\right\rangle. \tag{16}$$

We give $\boldsymbol{K}'$ equal to $\boldsymbol{K}$ so that $\boldsymbol{k}-\boldsymbol{G}'=\boldsymbol{K}$, where $\boldsymbol{G}'$ is also a reciprocal lattice vector, and obtain

$$\sum_{j\boldsymbol{K}}\left|\left\langle\psi_j^{(\boldsymbol{k})}\,\middle|\,\Psi_J^{(\boldsymbol{K})}\right\rangle\right|^2\delta(\epsilon-\epsilon_{\boldsymbol{K}J})=\frac{L}{l}\sum_{MN\boldsymbol{r}'}e^{\mathrm{i}\boldsymbol{k}\cdot(\boldsymbol{r}'-\boldsymbol{r}_0(N))}c_{MJ}^{(\boldsymbol{k}-\boldsymbol{G}')*}c_{NJ}^{(\boldsymbol{k}-\boldsymbol{G}')}\left\langle\phi_M^{\boldsymbol{0}}\,\middle|\,\phi_{n'(N)}^{\boldsymbol{r}'}\right\rangle\delta(\epsilon-\epsilon_{\boldsymbol{k}-\boldsymbol{G}',J}). \tag{17}$$

By the property of the delta function, the spectral function is finite only when ϵ is equal to some $\epsilon_{\boldsymbol{k}-\boldsymbol{G}',J}$, so the spectral weights can be naturally introduced as follows, except for the degenerate points about $\epsilon_{\boldsymbol{k}-\boldsymbol{G}',J}$.

$$\mathcal{A}_{\boldsymbol{k}J}=\frac{\bar{A}(\boldsymbol{k},\epsilon_{\boldsymbol{k}-\boldsymbol{G}',J})}{\delta(0)}=\frac{L}{l}\sum_{MN\boldsymbol{r}'}e^{\mathrm{i}\boldsymbol{k}\cdot(\boldsymbol{r}'-\boldsymbol{r}_0(N))}c_{MJ}^{(\boldsymbol{k}-\boldsymbol{G}')*}c_{NJ}^{(\boldsymbol{k}-\boldsymbol{G}')}\left\langle\phi_M^{\boldsymbol{0}}\,\middle|\,\phi_{n'(N)}^{\boldsymbol{r}'}\right\rangle. \tag{18}$$

For the case with degeneracy, we can also define $\mathcal{A}_{\boldsymbol{k}J}$ for the most right-hand side of Eq. 18 by mapping it to $\epsilon_{\boldsymbol{k}-\boldsymbol{G}',J}$. Moreover,

$$\sum_{\boldsymbol{r}'}\left|\phi_{n'(N)}^{\boldsymbol{r}'}\right\rangle e^{\mathrm{i}\boldsymbol{k}\cdot\boldsymbol{r}'}=\sum_{N'\boldsymbol{R}'}\delta_{n'(N),n''(N')}\left|\phi_{N'}^{\boldsymbol{R}'}\right\rangle e^{\mathrm{i}\boldsymbol{k}\cdot(\boldsymbol{r}_0(N')+\boldsymbol{R}')}, \tag{19}$$

and finally we obtain

$$\mathcal{A}_{\boldsymbol{k}J}=\frac{L}{l}\sum_{MN}c_{MJ}^{(\boldsymbol{k}-\boldsymbol{G}')*}c_{NJ}^{(\boldsymbol{k}-\boldsymbol{G}')}\sum_{N'\boldsymbol{R}'}\delta_{n'(N),n''(N')}e^{\mathrm{i}\boldsymbol{k}\cdot(\boldsymbol{R}'+\boldsymbol{r}_0(N')-\boldsymbol{r}_0(N))}\left\langle\phi_M^{\boldsymbol{0}}\,\middle|\,\phi_{N'}^{\boldsymbol{R}'}\right\rangle. \tag{20}$$

7. Visualization of allowed and forbidden bands in band dispersion of graphene nanoflakes

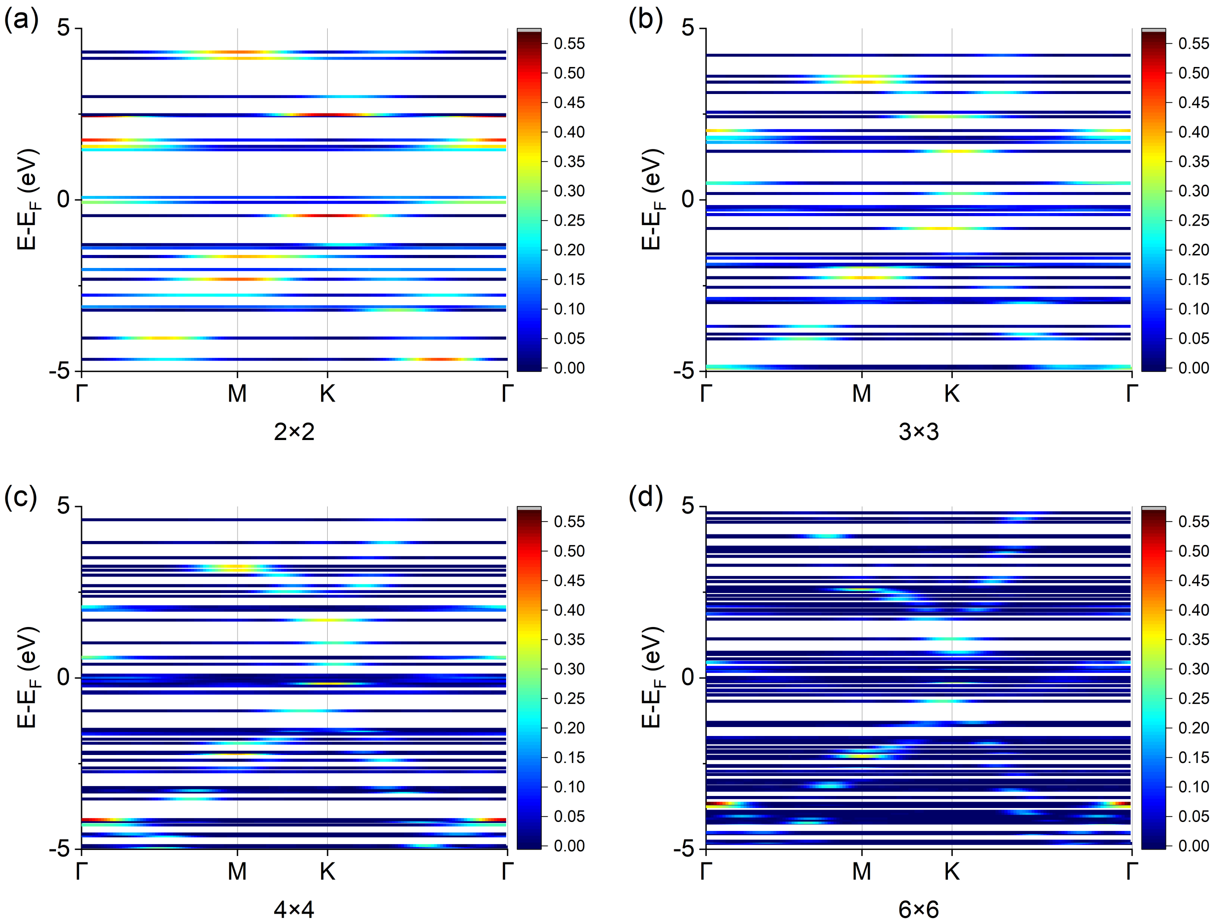

Figure S7: Band dispersion extracted from graphene nanoflake models of (a) 2×2, (b) 3×3, (c) 4×4, and (d) 6×6 rings, where the blank parts show domains that there are no MO energy levels, that is, the forbidden bands, and the colored parts show the allowed band domains whose colors stand for the spectral weight of the allowed bands. The k-path connects special k-points of $\Gamma(0, 0, 0)$, $M((2\pi/a)(1/2, 0, 0))$, and $K((2\pi/a)(2/3, 1/3, 0))$, where a is the lattice constant of pristine graphene. The Fermi energy is set to the origin of the energy axis.

8. Methods

Density functional calculations and unfolding analysis

We used the OpenMX code[15–17] (`http://www.openmx-square.org`) and performed first-principles calculations based on the DFT within the generalized gradient approximation (GGA)[18]. The following computational conditions were used to get the electronic structures for the band dispersion. Only the Γ-point was addressed to get the electronic structures.

For geometry optimization of 2×, 3 × 3, 4 × 4, and 6 × 6 graphene nanoflake models, the norm-conserving pseudopotentials we used included the 2s and 2p electrons for carbon as valence electrons. Cutoff energy values of 150 Ry was used for charge density. We also used a PAO basis set of carbon with the cutoff radius of 5.0 Bohr and the number of s- and p-orbital sets were 2 and 2, respectively. The vacuum width that avoid virtual interaction from image cells was set to 25 Å and more than 12 Å for out-of-plane and in-plane lattice constants for graphene flakes, respectively.

For graphene nanoflakes, we also used a PAO basis set of carbon with the cutoff radius of 6.0 Bohr and the number of s-, p-, and d-orbital sets were 2, 2, and 1, respectively, while the PAOs for hydrogen consisted of the 2 s-orbital and 1 p-orbital sets with the cutoff radius of 6.0 Bohr. The norm-conserving pseudopotentials we used included the 2s and 2p electrons and the 1s electrons for carbon and hydrogen as valence electrons, respectively. Cutoff energy values of 300 Ry was used for charge density. The vacuum width that avoid virtual interaction from image cells was set to 25 Å and more than 12 Å for out-of-plane and in-plane lattice constants for graphene flakes, respectively.

For a WS_2 nanoflake, we also used a PAO basis set of tungsten with the cutoff radius of 7.0 Bohr and the number of s-, p-, and d-orbital sets were 3, 2, and 2, respectively, while the PAOs for sulfur consisted of the 2 s-orbital, 2 p-orbital, and 1 d-orbital sets with the cutoff radius of 7.0 Bohr. We used the norm-conserving pseudopotentials including the 3s and 3p, and 5s, 5p, and 5d electrons for sulfur and tungsten as valence electrons, respectively. Cutoff

energy values of 300 Ry was used for charge density. The vacuum width was set to more than 28 Å in the out-of-plane and in-plane direction (see also section 3).

For a Bi/Ag(111)-($\sqrt{3} \times \sqrt{3})R30^{\circ}$ surface alloy nanoflake, we also used a PAO basis set of bismuth with the cutoff radius of 8.0 Bohr and the number of s-, p-, and d-orbital sets were 3, 2, and 1, respectively, while the PAOs for silver consisted of the 3 s-orbital, 2 p-orbital, and 1 d-orbital sets with the cutoff radius of 7.0 Bohr. We used the norm-conserving pseudopotentials including the 5d, 6s, and 6p, and 4p, 4d, and 5s electrons for bismuth and silver as valence electrons, respectively. Cutoff energy values of 220 Ry was used for charge density. The vacuum width was set to more than 33 Å in the out-of-plane and in-plane direction (see also section 4).

Non-collinear DFT with two-component spinor wavefunctions[19,20] was used to consider spin-resolved band unfolding, and spin-orbit interaction was taken into account in a fully relativistic treatment of the total angular momentum-dependent pseudopotential[21]. γ was set to the ratio of the total number of carbon atoms in the flake to the number of carbon atoms in the primitive cell of pristine graphene. In the tungsten disulfide and bismuth/silver surface alloy cases, γ was set to the ratio of the total number of atoms in the flake to number of atoms in the primitive cell.

Data Availability

The source code and data for giant molecule band unfolding (GMBU) can be found at https://doi.org/10.5281/zenodo.17815785.

References

(1) Davenport, J. W.; Watson, R. E.; Weinert, M. Linear augmented-Slater-type-orbital method for electronic-structure calculations. V. Spin-orbit splitting in Cu_3Au. *Phys. Rev. B* **1988**, *37* (17), 9985–9992. https://doi.org/10.1103/PhysRevB.37.9985.

(2) Boykin, T. B.; Klimeck, G. Practical application of zone-folding concepts in tight-binding calculations. *Phys. Rev. B* **2005**, *71* (11), 115215. https://doi.org/10.1103/PhysRevB.71.115215.

(3) Ku, W.; Berlijn, T.; Lee, C.-C. Unfolding First-Principles Band Structures. *Phys. Rev. Lett.* **2010**, *104* (21), 216401. https://doi.org/10.1103/PhysRevLett.104.216401.

(4) Qi, Y.; Rhim, S. H.; Sun, G. F.; Weinert, M.; Li, L. Epitaxial Graphene on SiC(0001): More than Just Honeycombs. *Phys. Rev. Lett.* **2010**, *105* (8), 085502. https://doi.org/10.1103/PhysRevLett.105.085502.

(5) Allen, P. B.; Berlijn, T.; Casavant, D. A.; Soler, J. M. Recovering hidden Bloch character: Unfolding electrons, phonons, and slabs. *Phys. Rev. B* **2013**, *87* (8), 085322. https://doi.org/10.1103/PhysRevB.87.085322.

(6) Lee, C.-C.; Yamada-Takamura, Y.; Ozaki, T. Unfolding method for first-principles LCAO electronic structure calculations. *J. Phys. Condens. Matter* **2013**, *25* (34), 345501. https://doi.org/10.1088/0953-8984/25/34/345501.

(7) Kosugi, T.; Nishi, H.; Kato, Y.; Matsushita, Y. Periodicity-Free Unfolding Method of Electronic Energy Spectra. *J. Phys. Soc. Japan* **2017**, *86* (12), 124717. https://doi.org/10.7566/JPSJ.86.124717.

(8) Chen, M.; Weinert, M. Layer k-projection and unfolding electronic bands at interfaces. *Phys. Rev. B* **2018**, *98* (24), 245421. https://doi.org/10.1103/PhysRevB.98.245421.

(9) Mayo, S. G.; Yndurain, F.; Soler, J. M. Band unfolding made simple. *J. Phys. Condens. Matter* **2020**, *32* (20), 205902. https://doi.org/10.1088/1361-648X/ab6e8e.

(10) Dai, Z.; Jin, G.; He, L. First-principles calculations of the surface states of doped and alloyed topological materials via band unfolding method. *Comput. Mater. Sci.* **2022**, *213* (July), 111656. https://doi.org/10.1016/j.commatsci.2022.111656.

(11) Chaurasiya, R.; Dixit, A.; Pandey, R. Strain-mediated stability and electronic properties of WS2, Janus WSSe and WSe2 monolayers. *Superlattices Microstruct.* **2018**, *122* (May), 268–279. https://doi.org/10.1016/j.spmi.2018.07.039.

(12) Yamaguchi, N.; Kotaka, H.; Ishii, F. First-principles study of Rashba effect in ultra-thin bismuth surface alloys. *J. Cryst. Growth* **2017**, *468*, 688–690. https://doi.org/10.1016/j.jcrysgro.2016.09.075.

(13) Bian, G.; Wang, X.; Miller, T.; Chiang, T.-C. Origin of giant Rashba spin splitting in Bi/Ag surface alloys. *Phys. Rev. B* **2013**, *88* (8), 085427. https://doi.org/10.1103/PhysRevB.88.085427.

(14) Ast, C. R.; Henk, J.; Ernst, A.; Moreschini, L.; Falub, M. C.; Pacilé, D.; Bruno, P.; Kern, K.; Grioni, M. Giant Spin Splitting through Surface Alloying. *Phys. Rev. Lett.* **2007**, *98* (18), 186807. https://doi.org/10.1103/PhysRevLett.98.186807.

(15) Ozaki, T. Variationally optimized atomic orbitals for large-scale electronic structures. *Phys. Rev. B* **2003**, *67* (15), 155108. https://doi.org/10.1103/PhysRevB.67.155108.

(16) Ozaki, T.; Kino, H. Numerical atomic basis orbitals from H to Kr. *Phys. Rev. B* **2004**, *69* (19), 195113. https://doi.org/10.1103/PhysRevB.69.195113.

(17) Ozaki, T.; Kino, H. Efficient projector expansion for the ab initio LCAO method. *Phys. Rev. B* **2005**, *72* (4), 045121. https://doi.org/10.1103/PhysRevB.72.045121.

(18) Perdew, J. P.; Burke, K.; Ernzerhof, M. Generalized gradient approximation made simple. *Phys. Rev. Lett.* **1996**, *77* (18), 3865–3868. https://doi.org/10.1103/PhysRevLett.77.3865.

(19) Barth, U. von; Hedin, L. A local exchange-correlation potential for the spin polarized case. i. *J. Phys. C Solid State Phys.* **1972**, *5* (13), 1629–1642. https://doi.org/10.1088/0022-3719/5/13/012.

(20) Kubler, J.; Hock, K.-H.; Sticht, J.; Williams, A. R. Density functional theory of non-collinear magnetism. *J. Phys. F Met. Phys.* **1988**, *18* (3), 469–483. https://doi.org/10.1088/0305-4608/18/3/018.

(21) Theurich, G.; Hill, N. A. Self-consistent treatment of spin-orbit coupling in solids using relativistic fully separable ab initio pseudopotentials. *Phys. Rev. B* **2001**, *64* (7), 073106. https://doi.org/10.1103/PhysRevB.64.073106.
